# Early access to science research opportunities: Growth within a geoscience summer research program for community college students

**DOI:** 10.1371/journal.pone.0293674

**Published:** 2023-12-21

**Authors:** Christine Okochi, Anne U. Gold, Alicia Christensen, Rebecca L. Batchelor

**Affiliations:** Cooperative Institute for Research in Environmental Science, University of Colorado Boulder, Boulder, Colorado, United States of America; Institute of Medical Biochemistry Leopoldo de Meis (IBqM) - Federal University of Rio de Janeiro (UFRJ), BRAZIL

## Abstract

Undergraduate research experiences benefit students by immersing them in the work of scientists and often result in increased interest and commitment to careers in the sciences. Expanding access to Research Experience for Undergraduate (REU) programs has the potential to engage more students in authentic research experiences earlier in their academic careers and grow and diversify the geoscience workforce. The Research Experience for Community College Students (RECCS) was one of the first National Science Foundation (NSF)-funded REU programs exclusively for 2-year college students. In this study, we describe findings from five years of the RECCS program and report on outcomes from 54 students. The study collected closed- and open-ended responses on post-program reflection surveys to analyze both student and mentor perspectives on their experience. Specifically, we focus on students’ self-reported growth in areas such as research skills, confidence in their ability to do research, and belonging in the field, as well as the mentors’ assessment of students’ work and areas of growth, and the impact of the program on students’ academic and career paths. In addition, RECCS alumni were surveyed annually to update data on their academic and career pursuits. Our data show that RECCS students learned scientific and professional skills throughout the program, developed a sense of identity as a scientist, and increased their interest in and excitement for graduate school after the program. Through this research experience, students gained confidence in their ability to “do” science and insight into whether this path is a good fit for them. This study contributes to an emerging body of data examining the impact of REU programs on community college students and encourages geoscience REU programs to welcome and support more community college students.

## Introduction

Research experiences provide undergraduate students exposure to and immersion in the work of scientists and are one way to increase participation and pursuit of geoscience careers and degrees. In 2013, the National Science Foundation (NSF) broadened the target population of their competitive research experience for undergraduate (REU) programs from rising juniors and seniors at four-year colleges to also include community college students and high school students. Recruiting students earlier in their educational career can influence students’ career trajectories and is a promising strategy to increase the pool of talented and diverse students interested in the geosciences, an important goal in geoscience education and for the geosciences as a whole [1]. The geoscience field, which includes earth, atmosphere, ocean, and polar sciences, has recently shifted from viewing career paths as a (leaky) pipeline [2] towards a pathway framework [3] and has been illustrated by the braided river metaphor [4]. In embracing careers as a braided river with many pathways that lead towards a geoscience career, REU programs have the potential to open channels for community college students and inspire them to select a geoscience field. Research shows that only 20% of geoscience majors started college with the intent to major in a geoscience field [5]—rather these fields are often “discovered” in college [6]. Considering that over a third of all undergraduate students in the U.S. (39%) are enrolled in community colleges, including many from groups historically underrepresented in the sciences [7], early exposure to the geosciences, including opportunities to immerse in geoscience research through short-term research experiences, provides important opportunities to engage students and grow and diversify the geoscience workforce.

The 2018 National Academy of Sciences report on indicators for monitoring undergraduate science, technology, engineering, and math (STEM) education highlighted the need to involve students in authentic STEM practices as well as the importance of supporting transfer from two-year to four-year colleges [8]. Specifically, the report describes three important positive outcomes from REU programs: *1) increased retention and persistence of students in STEM*, *2) cognitive outcomes such as promotion of STEM disciplinary knowledge and practices*, *and 3) integration of students into STEM culture or affective outcomes*. The Research Experience for Community College Students (RECCS) was one of the first REU programs supported by the NSF that exclusively engaged community college students. Here we explore the impact of an REU program in which community college students were matched with geoscience faculty mentors at a university or national lab for a summer research experience. The program offered students both exposure to authentic STEM research and provided support for a two-year to four-year college transfer.

In this paper, we describe program findings and student reflections from across five years of the RECCS program and report on outcomes from 54 community college students. We examine data through the lens of the following research question: *In which ways does participation in the RECCS program impact community college students’ preparedness for a career in the sciences and their academic career trajectories*? Specifically, we focused on students’ self-reported growth in areas such as research skills, confidence in their ability to do research, and belonging in the field; the mentors’ assessment of students’ work and areas of growth; and the impact of the program on students’ academic and career paths.

### Background

The Council on Undergraduate Research defines an undergraduate research experience as “A mentored investigation or creative inquiry conducted by undergraduates that seeks to make a scholarly or artistic contribution to knowledge” [9]. Research experiences are implemented as multi-week summer research programs, ongoing research programs throughout the year, or course-based programs [10]. The National Science Foundation describes research experience programs as “one of the most effective avenues for attracting students to and retaining them in science and engineering, and for preparing them for careers in these fields” [11]. Many studies have explored the success factors and benefits to students that are outlined in the National Academy’s report on research experiences [8]. Results of previous studies indicate that participants in research experiences were more likely to enroll in and complete a STEM major and more likely to continue toward a graduate degree, e.g., [12–15]. Students often reported that their participation in a research experience confirmed their interest in a STEM career path and that research experience formed a stepping-stone into a STEM career, e.g., [16–18]. Prior studies also described a wide range of skill and knowledge gains ranging from disciplinary content knowledge that allowed students to situate a research question in their field of study, to the development of research skills such as data collection and analysis in laboratory and field studies, e.g., [16–19]. Studies further described that students developed an overall understanding of the scientific process, including the reading of primary scientific literature; and intellectual skills such as working collaboratively, critical thinking, leadership, and scientific communication, e.g., [16–20]. Finally, prior studies described REU programs as formative for the development of students’ identities as scientists and a sense of belonging to the scientific community [21–23]. Over the course of their research experiences students developed relationships with their mentors, intellectually engaged with the topic, developed ownership of the project, and learned how to overcome hurdles, e.g., [16, 17, 19, 24]. All these positive outcomes resulted in students feeling part of the STEM community and developing self-confidence around independent research, which in turn often resulted in interest in or commitment to the discipline [25, 26]. Participation in an REU program also allowed students to initiate a professional network of scientists, starting with their peers, mentors, members of their research group, or the REU program staff.

Initiatives to overcome barriers to engaging community college students in these research opportunities, as described by Hewlett [27], and expand access to research opportunities at community colleges are gaining momentum [28]. And there is a growing body of evidence of the positive impacts REUs have on community college students, e.g., [29–32]. This study contributes to the emerging body of data, examining the impact of an REU program on community college students in the geosciences in particular, and best practices for welcoming and supporting more community college students in geoscience REU programs.

### Theoretical framework

The theoretical framework underpinning the learning process that occurs during summer research experiences builds on Vygotsky’s social constructivism [24, 33]. A social constructivist approach emphasizes that learning is a continuous process in which knowledge is constantly reconstructed and new knowledge is integrated with prior knowledge [34]. In REU programs, students share their understanding and views of science concepts with their mentors and research groups, other REU students, and REU program staff, using the language of the scientific community, reflecting a common understanding of the scientific community, and demonstrating skills that are used by scientists. Thus, students learn science in an authentic research setting, while they are embedded in the social context of a research lab or group, and through interactions with scientists. As the research mentor engages with the student in sharing knowledge, working through challenges, and discussing the science, social constructivism proposes that students learn and problem-solve beyond their knowledge level. This fosters critical thinking and results in learners that are motivated and independent [33]. Hunter et al. [24] further described the extension of the social constructivist pedagogical approach into a learning model that builds on communities of practice, in which newcomers (the students) are socialized into the practice of the community of scientific research, through mutual engagement with, and direction and support from, experienced scientists. Thus, students learn how to conduct research.

The design of the RECCS program follows the social constructivist approach and community of practice framework, as student researchers learn about scientific research in an authentic setting and are paired with research mentors that introduce them to the research culture and provide the context in which students develop into independent researchers. RECCS students develop their own research questions under the guidance of their mentors and are encouraged to relate their findings to the “big picture” in the context of their research group and the broader field. As their projects unfold, students often find the need to revise their questions in response to unexpected results or unforeseen interruptions in their original research plan. Thus, students expand their knowledge of their particular research topic, as well as the research process, or what a scientist does. The RECCS student cohort, peer mentors, and the RECCS program staff form an additional layer of mentors who guide the student in integrating newly learned knowledge with existing knowledge.

### Description of the RECCS program

RECCS is an NSF-funded REU program in environmental and geosciences that was designed for community college students in Colorado in consultation with local community college faculty members. RECCS students participate in a nine-week paid summer program where they complete an authentic research project guided by a research mentor or mentor team from research institutions such as the University of Colorado Boulder (CU Boulder), the National Oceanic and Atmospheric Administration (NOAA), and the U.S. Geological Survey (USGS). Throughout the summer, RECCS students work towards two program deliverables, a scientific poster and a short scientific oral presentation, about their individual research projects.

The RECCS program staff supports the student researchers individually and as a cohort, complementing the support from research mentors and striving to create an inclusive learning community. Students spend an introductory week with the RECCS program staff learning foundational research skills such as asking research questions, reading scientific papers, interpreting graphs, and exploring what makes a good poster and presentation. Throughout the program they also reflect on the scientific process and the thinking and working like a scientist to foster students’ science identity and feeling of belonging within science. The introductory week also includes an overnight trip to the University of Colorado’s Mountain Research Station, a field station in the nearby Rocky Mountains, for cohort-building and hands-on fieldwork activities. For the duration of the program, students meet as a cohort with the RECCS program team once a week for a science communication and professional development workshop. Weekly workshop assignments are scaffolded to keep students moving toward the program deliverables and to reflect the scientific process. For example, as students dive into background reading early in the summer, they learn how to draft an introduction to their project. Professional development sessions include presentations from scientists, career panels, training on science ethics, resume writing workshops, and goal setting. These weekly cohort check-ins also allow students to share their current challenges or achievements and to support one another. RECCS alumni provide further support, serving as peer mentors that help students to understand their experience through a peer lens. After the program, RECCS students are invited to join an alumni email list and an alumni LinkedIn group to stay connected and receive information about future professional opportunities such as internships, fellowships, or jobs.

The RECCS application process is competitive, with an average of 50 applicants per year for 10 summer researcher positions. The RECCS program criteria require that students are enrolled in a Colorado community college and have not previously earned a college degree. While applicants need to demonstrate interest and some coursework in STEM, the RECCS selection committee takes a holistic view of student potential, focusing their selection process on students’ responses to several open-ended application questions (e.g., potential benefit of the program, an example of overcoming a challenge in a previous work experience, future academic and career goals). This gives students who may be early in their science education, who may not yet have had exposure to the geosciences, or who may be pursuing a second career an opportunity to describe other experiences and assets they bring to the program that might not be represented in traditional metrics, such as courses taken or GPA, alone.

### Study participants

This study includes data from five RECCS cohorts (2015–2019) for a total of 54 students. Participants had varied backgrounds and prior experiences. Forty-one percent (41%) were first-generation college students, which is higher than the national average for community colleges of 29% [7]. Although age was not asked of students in the 2015–2017 cohorts, in the 2018 and 2019 cohorts 39% were considered non-traditional students, defined by NSF as 30 years old or older. (See Table 1 for a summary of student demographics). Finally, at the start of the program, 50 participants (93%) were enrolled in STEM disciplines at their community colleges and four (7%) were enrolled in non-STEM disciplines.

**Table 1:** Study participants (N = 54 students).

**Student characteristics**	
First-generation college students	41%
Non-traditional students (n = 20)	39%
Rural college students	13%
Military veterans	6%
**Race and ethnicity**	
White	69%
More than one race or ethnicity selected	24%
Asian	4%
African American or Black	2%
Hispanic or Latinx	2%
**Gender**	
Female	59%
Male	41%

This study also includes data from 41 research mentors, 11 of whom participated more than one summer and thus provided assessment of more than one student in the study. Seventy-six percent of mentors were university-based researchers while 24% were affiliated with a national research lab (i.e., NOAA, USGS) at the time of participation. Most mentors (approximately 70%) worked with another mentor as part of a mentor team, typically made up of a research faculty mentor and a graduate student mentor, while the remainder mentored students individually.

## Study design and methods

The study collected quantitative and qualitative data in the form of closed- and open-ended responses on post-program reflection surveys to analyze both student and mentor perspectives on their experiences. In addition, RECCS alumni were surveyed annually to update data on their academic and career pursuits. This study was approved by the University of Colorado Boulder Institutional Review Board Protocol 15–0034. Students and mentors were recruited to the study at the start of each summer (2015–2019). All 54 students and 41 mentors included in the study signed written consent forms. Authors replaced names with identifiers after data collection.

### Student reflection survey

The student reflection survey included intact item blocks from the Undergraduate Research Student Self-Assessment (URSSA) survey, a widely used and validated instrument to assess student outcomes of undergraduate research experiences in the sciences [35]; please see S1 File for URSSA questions included in the student survey. For the first three URSSA question blocks, students self-reported how much personal gain they perceived in three areas: i) research skills, ii) thinking and working like a scientist, and iii) personal and professional gains related to research, on a 5-point Likert scale from *no gains* to *great gains*. The question block on research skills included items like *Explaining my project to people outside my field; Conducting observations in the field or lab;* and *Calibrating instruments*. The question block about thinking and working like a scientist included items such as *Analyzing data for patterns* and *Problem-solving in general*. Items assessing personal and professional gains included *Confidence in my abilities* and *Comfort collaborating in a group*. A fourth URSSA question block asked students to report how much time they spent engaged in eight different behaviors typical of a researcher, for example, *Engage in real-world science research; Feel responsible for the project*; and *Feel part of a scientific community*. Students self-reported their engagement in these behaviors on a 5-point Likert scale from *none* to *a great deal*. Following Weston & Laursen [26], a mean value was calculated for each of these four URSSA question blocks.

A final URSSA survey question block was included in the student survey to collect reflections on how the program impacted their academic or professional interests and preparedness. This question included eight statements, such as *My research has prepared me for graduate school* and *Doing research introduced me to a new field of study*, rated on a 4-point Likert scale from *Strongly disagree* to *Strongly agree*. To add context to this survey question, students were also asked an open-ended follow-up question to elaborate on how the research experience may have influenced their thinking about future career and graduate school plans.

### Mentor reflection survey

At the end of the program, mentors were asked to rate students on several aspects of the research experience, such as preparation, work ethic, and quality of deliverables, using a 5-point Likert scale from *Well below average* to *Well above average* in terms of mentors’ expectations for undergraduate researchers; please see S1 File for questions included in the mentor survey. For students who were mentored by a mentor team, their mentors’ ratings were averaged so that there was one rating per student. During analysis, mentor responses for the two highest ratings–*Well above average* and *Above average*–were collapsed into one rating for *Above average*. Likewise, mentor responses for the two lowest ratings–*Below average* and *Well below average*–were collapsed into one rating for *Below average*.

Starting with the 2018 cohort, mentors also rated students’ progress as scientists-in-training on a 4-point Likert scale from *Very little* to *Significant*. As with the previous mentor survey question, for students who were mentored by more than one person, an average of the mentors’ ratings was used in the analysis. Finally, mentors were asked to briefly elaborate on areas in which their mentees made the most progress.

### Data analysis

Data from student and mentor surveys were used to substantiate findings from both the student and mentor perspectives. Closed-ended, Likert-scale survey responses were reported using descriptive statistics. Open-ended responses were coded using content analysis [36] and following a hybrid approach that included both deductive and inductive coding [37]. In the student survey, after responding to the closed-ended Likert-scale question about the program’s impact on their academic or professional interests and preparedness (see above), students were asked to elaborate on how the research experience might have influenced their future plans. Taking a hybrid approach to coding these responses, first, a set of eight a priori codes was created based on the content of the eight statements in the closed-ended Likert question that preceded the open-ended question, such as, *Prepared me for graduate school*, *Prepared me for a job*, *Introduced me to a new field of study*, and *Enhanced my resume*. During the coding process, six additional (a posteriori) codes were added to the coding scheme to account for responses that did not fit one of the existing (a priori) codes. For example, *Increased confidence* and *Still undecided* were added (see complete list of codes in the Results section). In the mentor survey, after responding to the closed-ended Likert-scale question described above about student progress as scientists-in-training, mentors were asked to briefly elaborate on the areas students made the most progress. To analyze these responses, a set of a priori codes was created based on the content in the four URSSA question blocks (i.e., *Research skills; Thinking and working like a scientist; Personal and professional gains;* and *Engaged in the behaviors of a researcher*). During coding, one additional code was added a posteriori: *Awareness of career options* (see the complete list of codes in Results section).

For reliability, two researchers independently coded all responses using the software Dedoose and Cohen’s kappa was calculated; please see S3 File for reliability data. To reconcile the five codes with lower interrater reliability scores (k<0.50) in the student data set, the two researchers met to refine the definition of those codes and re-apply them to the responses. Final inter-rater reliability for each code ranged from k = 0.51 to 0.84. Reliability for the coded mentor responses was high for all codes (k = 0.92) and did not require further discussion. Code frequencies for the student and mentor open-ended responses were reported in frequency tables.

### Tracking alumni academic and career paths

Data on academic enrollment and professional positions of RECCS alumni were collected through an annual alumni survey sent by email to all RECCS alumni. To supplement the surveys, we mined data from the RECCS LinkedIn group, which was a valuable tool for keeping current with alumni who may not have returned the survey.

## Results

### Finding 1: RECCS students reported gains in research skills; growth in thinking like scientists; and personal gains related to research, such as increased confidence. Students also felt engaged in the real work of scientists during their research experience

**Fig 1:**
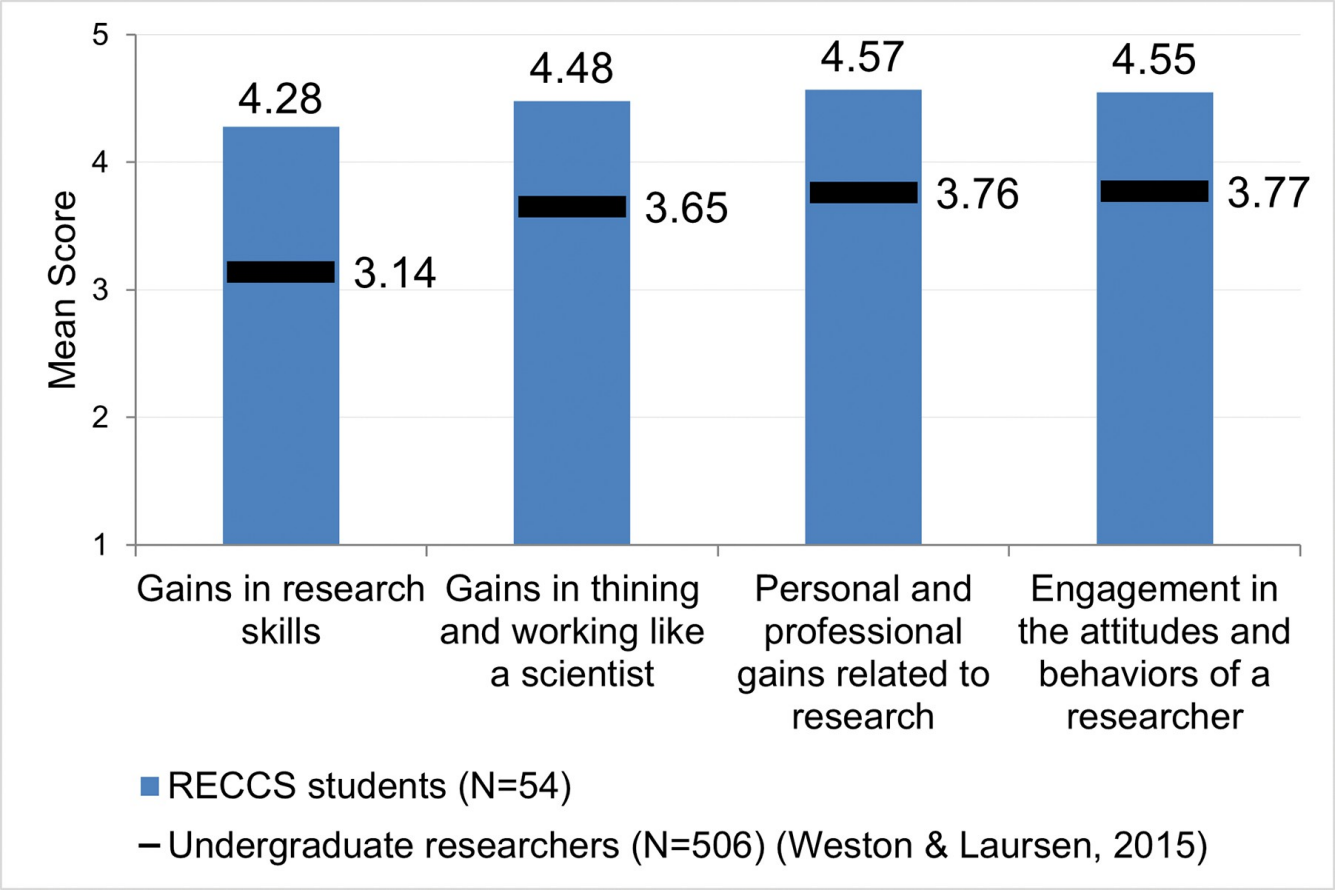
Self-reported data at the end of the research experience. RECCS student responses are compared with responses from undergraduate researchers in a large sample [26].

Results from the student reflection survey show that as a group, RECCS students reported good gains in all categories that were assessed with respect to engaging in scientific work–*Research skills; Thinking and working like a scientist;* and *Personal and professional gains related to research*. Gains were reported on a 5-point scale with mean scores ranging from 4.28 to 4.57 for these three question blocks (Fig 1); see S2 File for complete survey data. Students also reported on their time spent engaged in the attitudes and behaviors of a researcher on a 5-point scale. RECCS students felt that they engaged in these behaviors a fair amount of the time during their research experience, a mean of 4.55 for this question block. Compared to the mean scores from a large sample of undergraduate student researchers (n = 506) published by Weston and Laursen [26], RECCS students self-reported higher average gains and time spent engaged in the behaviors of a researcher.

Looking at each item in these question blocks, RECCS students reported the most growth in *Preparing a scientific poster* (4.78), a research skill that was explicitly taught during the RECCS workshop (Table 2). However, students also reported high growth in areas not directly taught as part of their cohort training but that they experienced in their daily work within their research groups. For example, students gained *Confidence in my ability to contribute to science* (4.76), *Confidence in my ability to do research* (4.74), *Confidence in my ability to do well in future science courses* (4.60), and *An understanding of what everyday research is like* (4.73). These data suggest that students felt better equipped with newly acquired skills, confidence for pursuing scientific research further, and an awareness of what that pursuit entails. The specific attitudes and behaviors of a researcher that students reported engaging in most frequently during the summer were *Feeling responsible for the project* (4.78), *Engaging in real-world research* (4.74), *Feeling like a scientist* (4.63), *Feeling part of the scientific community* (4.62), and *Thinking creatively about the project* (4.62). These data suggest that students were developing a sense of belonging to the scientific community.

**Table 2:** Self-report data for each item in the URSSA question blocks for RECCS students from 2015–2019 (N = 54). Responses on a scale of 1 to 5; data reported as group mean.

**Gains in research skills**	
Writing scientific reports or papers.	4.24
Making oral presentations.	4.47
Defending an argument when asked questions.	4.14
Explaining my project to people outside my field.	4.71
Preparing a scientific poster.	4.78
Keeping a detailed lab notebook.	4.05
Conducting observations in the lab or field.	4.19
Using statistics to analyze data.	4.33
Calibrating instruments needed for measurement.	3.77
Working with computers.	4.38
Understanding journal articles.	4.22
Conducting database or internet searches.	4.12
Managing my time.	4.27
Mean for question block	4.28
**Gains in thinking and working like a scientist**	
Analyzing data for patterns	4.47
Figuring out the next step in a research project	4.51
Problem-solving in general.	4.47
Formulating a research question that could be answered with data.	4.45
Identifying limitations of research methods and designs.	4.64
Understanding the theory and concepts guiding my research project.	4.49
Understanding the connections among scientific disciplines.	4.38
Understanding the relevance of research to my coursework.	4.43
Mean for question block	4.48
**Personal and professional gains related to research work**	
Confidence in my ability to do research.	4.74
Confidence in my ability to contribute to science.	4.76
Comfort in discussing scientific concepts with others.	4.44
Comfort in working collaboratively with others	4.57
Confidence in my ability to do well in future science courses.	4.60
Ability to work independently	4.33
Developing patience with the slow pace of research.	4.46
Understanding what everyday research work is like.	4.73
Taking greater care in conducting procedures in the lab or field.	4.41
Mean for question block	4.57
**Engage in attitudes and behaviors of a researcher**	
Engage in real-world science research.	4.74
Feel like a scientist.	4.63
Think creatively about the project.	4.62
Try out new ideas or procedures on your own.	4.25
Feel responsible for the project.	4.78
Work extra hours because you were excited about the research.	4.35
Interact with scientists from outside your school.	4.42
Feel part of a scientific community.	4.62
Mean for question block	4.55

Of these four URSSA constructs, students reported the least gains in research skills (4.28), and particularly for the items *Calibrating instruments needed for measurement* (3.77) and *Keeping a detailed notebook* (4.05). On a reflective post-survey, it is difficult to interpret a response of no or little gain without the context of students’ skill level before the program. However, lower gains reported might suggest that not all students had the same opportunity to engage in practicing a particular skill during the nine weeks, perhaps due to the emphasis some mentors or research projects might have placed on other skills.

### Finding 2: Mentors reported that most students produced high-quality deliverables and made significant progress as scientists-in-training

**Fig 2:**
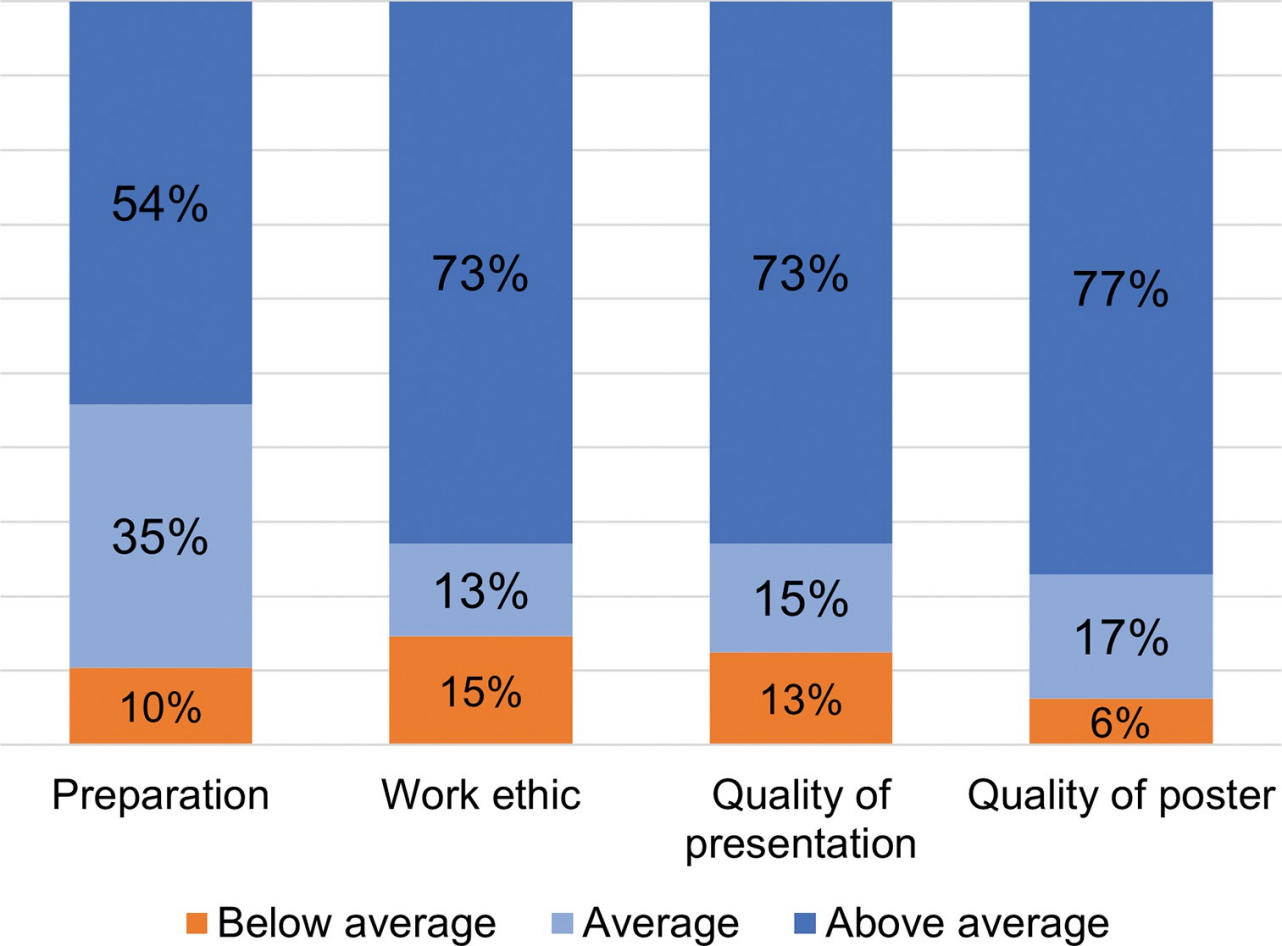
Mentor perceptions of student preparation, work ethic, and quality of deliverables in terms of their expectations for undergraduate researchers (N = 48 students rated by 63 mentors).

Most mentors assessed RECCS students’ preparation, work ethic, and the quality of their posters and presentations as *Average* or *Above average*, in terms of their expectations for undergraduate researchers (Fig 2); see S2 File for complete survey data. Approximately half of RECCS students (54%) were rated *Above average* in preparation and an even greater percentage, nearly three-quarters (73%), as *Above average* for work ethic. For quality of student deliverables, approximately three-quarters of their posters (77%) and final presentations (73%) were rated as *Above average*.

**Fig 3:**
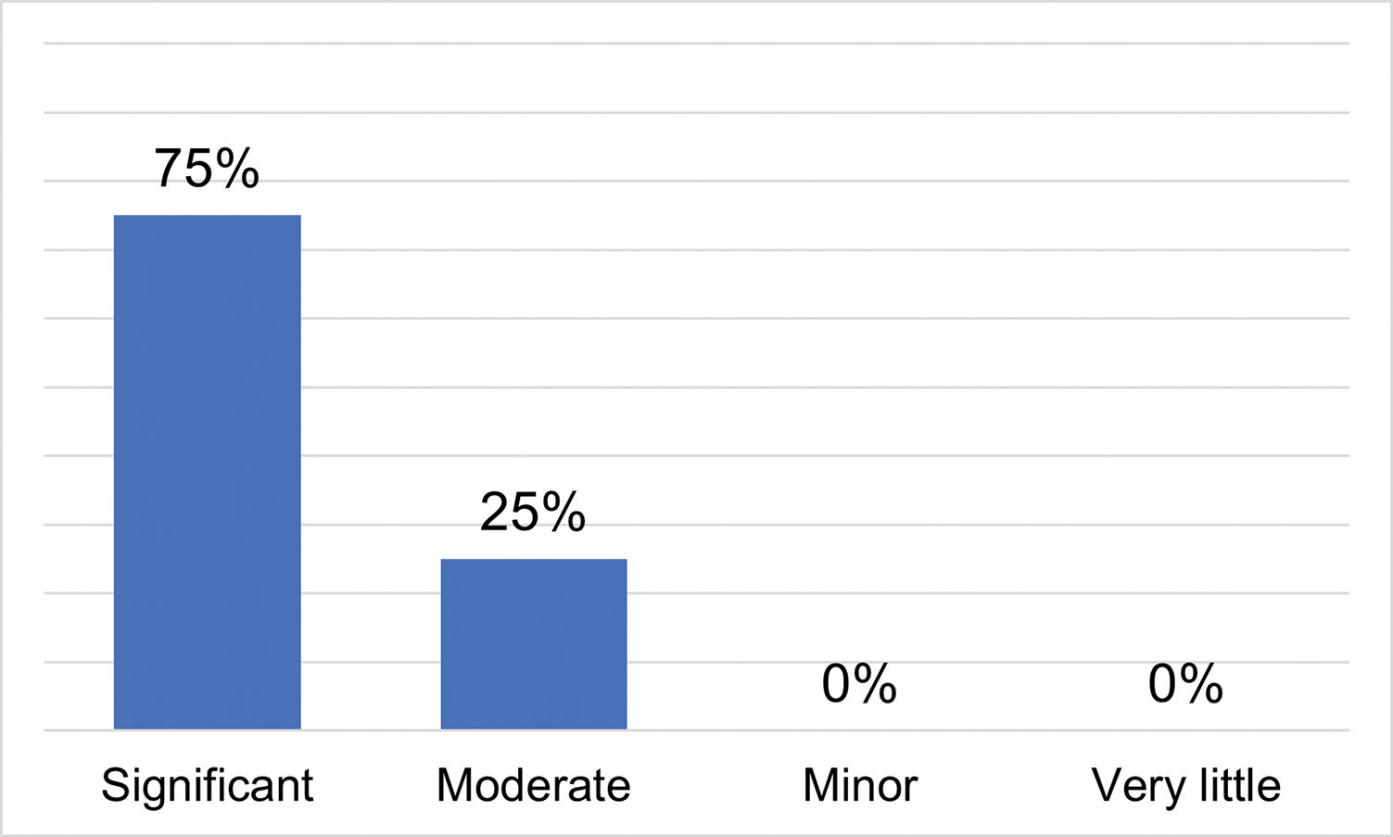
Mentor ratings of students’ progress as scientists-in-training (n = 20 students rated by 29 mentors*). *Data collected from 2018 and 2019 cohorts only.

Mentors in the 2018 and 2019 cohorts were also asked to rate students’ progress as scientists-in-training and reflect on the areas they thought students made the most progress. Twenty-nine mentors provided ratings of 20 students. Three-quarters of these students were rated as having made *Significant* progress as scientists-in-training during the research experience and the remaining one-quarter as having made *Moderate* progress (Fig 3): see S2 File for complete survey data.

When elaborating on areas of student progress, most mentors listed areas that fell under the code of research skills (82%), such as computer programming skills, lab/field skills, or science communication skills (Table 3). For example, one mentor described their mentee as *“Mastering basic microbiology lab skills that they had no prior experience with*, *and data analysis (they learned a coding language and were able to perform statistical analyses and also generate high-quality figures with little assistance*!*)”*. Another mentor reflected, *“She became much better at expressing why her research was important and in understanding how to express concepts without overstating them”*. Mentors also described how some students demonstrated thinking more like a scientist by the end of the summer (50%), which included things like critical thinking or gaining a better understanding of the scientific process. One mentor assessed their mentee as having made the most progress “*Problem-solving issues and coming up with ideas to test in general*.*”* Related to personal gains, mentors recognized a boost in some students’ confidence. For example, *“I think what was most important was the confidence she gained to work on things outside of her comfort zone”*. Of the same student, another mentor said, *“She expressed at the end that she was not used to being asked what she thought about things”*. Some mentors noted that their mentees gained awareness of what everyday research was like. For example, *“I think he learned a lot about what is really involved in research*, *and that not all of it is fieldwork or cool results–and there is lots of hard work too*. *I think that was valuable for him”*. Finally, one mentor’s observations of student growth included a greater awareness of career options, specifically understanding the different levels of academic degrees. Overall these mentor reflections on areas of student growth, while representing only a subset of students from the 2018 and 2019 cohorts, support the findings students self-reported.

**Table 3:** Percent of mentor responses that included content in each code category for the question, *In what areas did your student make the most progress*? (N = 28 mentors).

Code:	Code type:	Example(s):	Percent of responses:
Research skills	a priori	Conducting observations; keeping a lab notebook; communicating about their work; computer programming	82%
Thinking and working like a scientist	a priori	Analyzing data; figuring out the next steps in research; problem-solving in general	50%
Personal gains related to research work	a priori	Confidence in abilities; comfort collaborating as part of a team; understanding what day-to-day research is like	29%
Attitudes and behaviors of scientist	a priori	Interact with scientists outside your school (e.g., at a conference)	4%
Awareness of options	a posteriori	Better understanding of graduate school opportunities and resources	4%

### Finding 3: Students agreed that the research experience positively impacted their interest in and preparedness for graduate school

**Fig 4:**
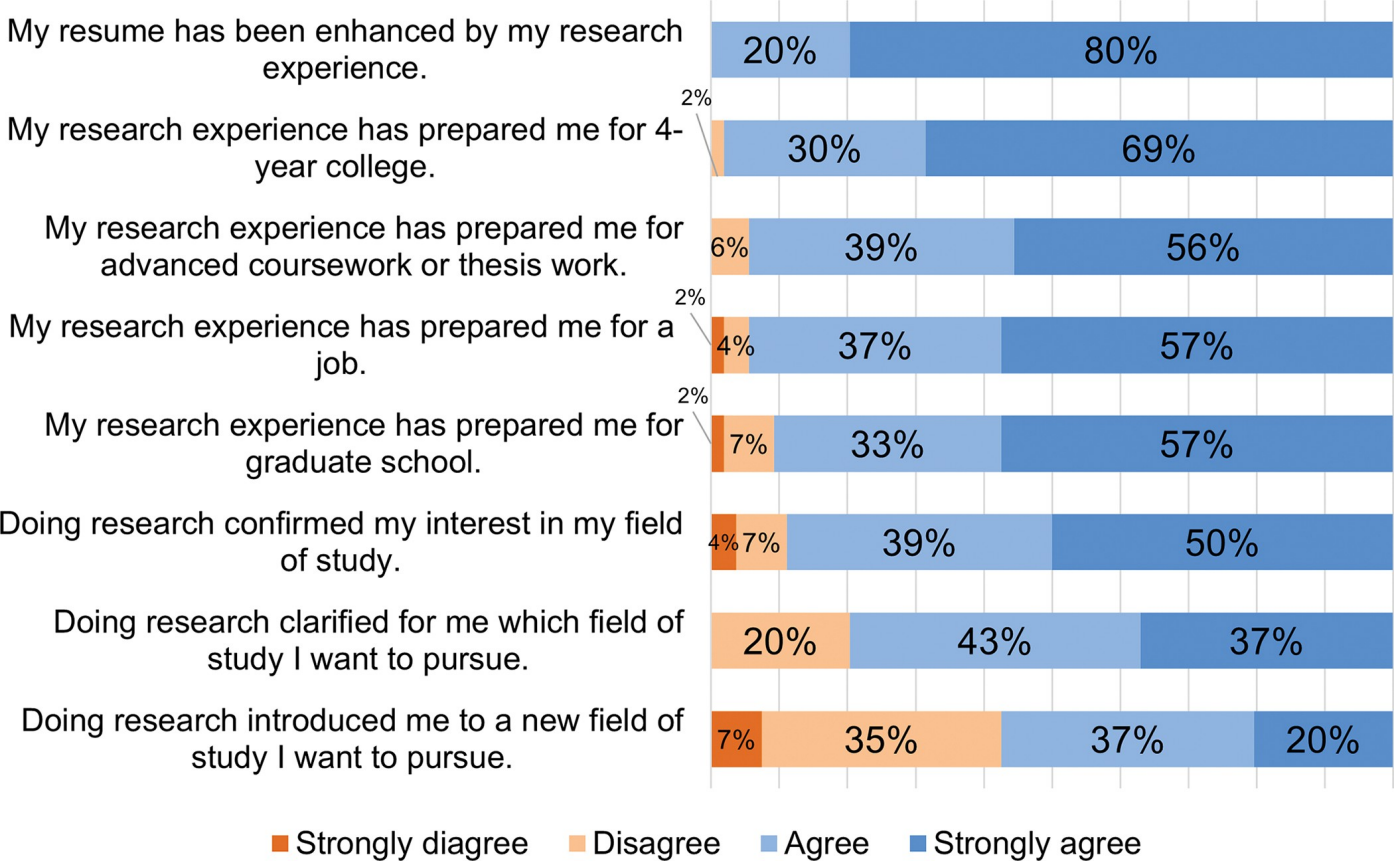
Impact of the research experience on academic and professional preparedness and interest (N = 54).

Upon completion of the research experience, students reflected on how it impacted their preparedness for and interest in future academic or career opportunities (Fig 4); see S2 File for complete survey data. All of them agreed that the research experience enhanced their resumes, and nearly all agreed that it helped prepare them for a 4-year college. There was a similarly high percentage of students (>90%) who felt it prepared them for advanced coursework, prepared them for a job, and prepared them for graduate school.

When elaborating on how the research experience may have influenced their thinking about their future academic and career plans, students described impacts that went beyond the eight statements listed in the closed-ended question that preceded (see Table 4). Some did say they felt better prepared for graduate school (19%), but more of them expressed a real interest in attending (56%) and some in conducting research more specifically (22%). They described feeling less intimidated and more comfortable in a research environment, realizing that graduate school was feasible, and seeing graduate school as a more attainable goal to achieve. Some credited their mentors and the graduate students they met for influencing their thinking (22%). For example, *“Seeing the bigger projects that my mentor and his cohorts were working on made me want to pursue that as a career*, *and talking to them about graduate school made it less intimidating and more of a feasible possibility”*. Some students felt the research experience also made them aware of more career options and of the resources available to them. For example, one student wrote, “*I now know my options*. *As a first-generation college student*, *sometimes I have difficulties finding the appropriate resources*. *Through this program*, *I was exposed to new people*, *new resources*, *and a richer understanding of graduate school*. *Cost has always been a concern of mine*, *but I know that it is not impossible*. *(Especially in the sciences*!!*)”*.

**Table 4:** Percent of student responses that included content in each code category for the question, *How did your research experience influence your thinking about future career and graduate school plans*? *Please explain*. (n = 54).

Code:	Type of code:	Percent of responses:
Confirmed or inspired new interest in graduate school.	a posteriori	56%
Confirmed or inspired new interest in research.	a posteriori	22%
Introduced me to others in the scientific community.	a posteriori	22%
Made me aware of more options and resources.	a posteriori	19%
Prepared me for graduate school.	a priori	19%
Clarified for me which field of study I want to pursue.	a priori	15%
Increased confidence in my abilities.	a posteriori	9%
Introduced me to a new field of study I want to pursue.	a priori	7%
Confirmed interest in my field of study.	a priori	6%
Still undecided.	a posteriori	4%
Prepared me for a job.	a priori	4%
Prepared me for a 4-year college.	a priori	2%
Prepared me for advanced coursework.	a priori	0%
Enhanced my resume.	a priori	0%

Other students elaborated on how the experience influenced their planned field of study. For example, it clarified their desired field (e.g., *“I realized I loved studying science and mathematics*!*”* and “*I think that doing the research in my field showed that I still wanted to pursue my original degree*. *It showed me that my interest for environmental sciences was minimal and my goal for planetary science remained the same”)*. Others were exposed to a new field of interest that changed their plans, like the student who was considering adding a minor in biochemistry. Finally, two students (4%) were admittedly undecided about their future plans and did not elaborate further.

So, where are RECCS alumni now? Since completing the REU, 53 alumni from this study have remained in touch with the program via the alumni survey or the LinkedIn group. To date, 29 alumni (54.7%) have completed a degree (Table 5), most of them (27 out of 29) in STEM fields. Following those 27 alumni from STEM degrees to STEM employment indicates that nearly half were employed in STEM fields at the time of this writing (13 out of 27, 48%).

**Table 5:** Current academic status of RECCS alumni (n = 53).

	Currently enrolled in a degree program	Highest degree completed (not currently enrolled)
Associate	5.7%	13.2%
Bachelors	28.3%	37.7%
Masters	7.5%	3.8%
PhD	3.8%	0.0%
**Total**	**45.3%**	**54.7%**

### Limitations

REU programs are intensive research experiences that serve small numbers of students. Thus, the ability to report statistical significance in findings is limited and we ground all quantitative data in qualitative findings. The number of students we are reporting on (N = 54) is fairly small but constitutes five years of program experience. We also want to stress that despite efforts to find the best mentor-mentee match, the experience of each of these 54 students was unique and each mentor-mentee pair experienced the research experience differently. In this study, we tried to identify the commonalities in experiences that students and mentors described but some individual experiences likely differed from the shared or universal patterns we report. While the broad program structure was the same across all five years, we slightly modified the program from year to year. For example, students stayed one night at the university’s Mountain Research Station in 2014–2017 but in 2018 we increased the stay to two nights, which may have allowed stronger bonds to develop within the cohort. Another example is that in one of the years, the students had a cohort Facebook group, in other years they didn’t, which may have led to different levels of communication. These small changes in program elements may have slightly changed the experience of students in different cohorts.

## Discussion

### Student growth throughout a summer research experience has many facets

The results from the RECCS program show the benefits and impacts of the research experience for the community college students in the program across many different aspects. We showed that RECCS students learned scientific and professional *skills* throughout the program, developed a sense *science identity* and that students described increased motivation, interest, and excitement for a STEM *career* path or graduate-level research after the program. Our data suggest that students felt their research project was relevant and inspired a sense of project ownership, and working in a supportive RECCS environment built a sense of belonging to this community of scientists. Students benefited from learning research and technical skills—such as lab techniques, computer programming, and data analysis—as well as soft skills such as communicating and presenting their work. The supportive cohort, weekly training, and regular check-ins by the RECCS staff in addition to the mentor support created an environment that fostered students’ self-efficacy, boosted their confidence in their own abilities, and created a sense of community.

While similar benefits to participants in a variety of research experiences have been described in the literature, criticism has been raised that limited evidence exists to measure the specific impact of REU programs on participating students [17]. Impact analysis and program evaluation in REU programs have usually been based on student reflection and self-report data as the primary data source, which is subject to response bias [38]. In this paper, we present data from mentor reflections in addition to student self-report data (see Hunter et al. [24] for a detailed discussion of this approach). We found that most RECCS mentors tended to focus their assessment of student growth on areas of research and technical skills, or critical thinking and problem-solving skills, however more than a quarter also noticed students’ personal gains, such as confidence and comfort in a research environment. In a separate study, we measured the skill gains around paper and graph reading of RECCS students using eye tracking technology and showed that student skills over the course of the nine-week program became more expert-like, corroborating the self-reported data of RECCS students and their mentors who describe they gained research skills [20]. We argue that along with these gains in skills, increased confidence and the ability of students to see themselves as scientists are critical components of RECCS students’ success. Both self-efficacy and science identity are important predictors of success and persistence in STEM, particularly for those from underrepresented groups [39, 40]. Self-report data presented here suggest gains in RECCS students’ self-efficacy and science identity. Based on findings from others who correlate career intention with science identity and self-efficacy [41], we interpret RECCS students’ gain in these areas as factors that contributed to their persistence in the sciences. Further work on the impact of science identity on career success in REU program contexts is necessary to confirm this correlation.

Other studies have shown that a cohort and a scaffolded program towards an academic achievement can provide a structure and safety net that allows learners to overcome the negative impact of imposter feelings or self-doubt [42] and that it is important for individuals to feel competent in order to maximize motivation, performance and well-being [43]. The structure of the RECCS program may have provided this structure that allowed participants to develop science identity by countering feelings of self-doubt. The weekly workshop assignments and check-ins, along with support from the RECCS staff, peer mentors, and the cohort likely influenced student success within the program. The ongoing alumni support and the network of like-minded students continue to provide this supportive structure as RECCS alums explore their career paths.

### Community college students show strong growth as researchers throughout summer REU program and the impacts of such a program is large

In the Braided River career path framework [4], the RECCS program is intentionally designed as a channel for community college students to access research experience early in their careers. The program provides Colorado community college students the chance to work closely with researchers and within research groups from a research-intensive university or national lab for a summer and provides a supportive and encouraging environment, as well as scaffolding toward a final poster and oral presentation. Students spent the majority of their time each week (30 or more hours) with their research mentor teams and about five to ten hours per week with the RECCS staff and their cohort. The time with the cohort and RECCS staff and especially the scaffolded structure of supporting students in developing their program deliverables throughout the duration of the program appeared to provide an important anchor for student success and complemented the work with the mentors. The growth reported by students and mentors also highlights the assets and experiences that students bring to the individual research project and their research groups. While about half of mentors thought that students came in with average or below-average preparation, mentors lauded the above-average work ethic of three-quarters of the students and the similarly excellent quality of the final products (i.e., poster and oral presentation). Through a research experience like RECCS early in their academic careers, students can gain confidence in their ability to “do” science and insight into whether this path is a good fit for them. The summer researchers embraced the opportunity and thrived in the program even though they were in the early stages of foundational coursework at their community colleges.

### Summer research experiences provide opportunities to test research as a career pathway early in students’ academic experiences

Research experiences provide an opportunity for participants to develop a vision for STEM careers early in their career path and a vision of themselves as researchers, through exposure to role models in their peer mentors and research mentors and through the experience of completing an independent research project [15, 44, 45]. Our data corroborate these findings that a summer research experience is a critical juncture in the career path of community college students and an important tributary in the Braided River career path model.

Despite this foray into research being exploratory for some students, most of them finished the summer with a clearer plan for their future academic and research pursuits and were proud of their accomplishments. Considering that nationwide only 14% of STEM-educated workers with bachelor’s degrees were employed in STEM jobs [46], RECCS alumni with a science degree have so far remained in the science workforce in greater numbers (48%).

As a Colorado-focused program, about half the RECCS cohort lived within 50 miles of the CU campus, which allowed mentors who had funding available to offer students a research assistant position for the following semester. The opportunity for students to continue working with their mentors in paid research assistant positions, transfer to local Colorado 4-year colleges, and sustain the relationships they made with their cohort illustrates the benefits of an intentionally designed, local program to support and inspire community college students’ transition to a four-year degree and beyond. On the alum survey, we heard from students who enrolled at a 4-year institution together with others from their cohort, continuing to provide support for each other through the transition and their college experience.

Over the last three years, the COVID-19 pandemic impacted some of the RECCS alumni who were not employed in STEM jobs. Several alumni described this in the most recent alumni survey, explaining how setbacks during the pandemic forced them to delay applying to graduate programs, to focus their resources on childcare, or to choose a job outside of science out of necessity, but RECCS alumni described being hopeful of and intending to return to STEM fields in the future. More data collected over the coming years as RECCS alumni complete degrees, return to school, or change jobs will support a better understanding of the impact of the pandemic on STEM career trajectories.

### RECCS student growth viewed through the lens of social constructivism

The multi-faceted growth exhibited by community college students during their participation in the RECCS program can be understood within the framework of Vygotsky’s social constructivism, which underscores the role of social interactions and collaborative learning in cognitive development of learners. Our findings imply that the RECCS program serves as an authentic platform where students engage in a dynamic process of knowledge co-construction. A key component of Vygotsky’s theory is the notion that learning is a continuous process of reconstructing knowledge, where new understandings are woven into existing understanding, a process that happens throughout the mentor-mentee interactions and is supported by the weekly trainings. Our findings show that RECCS students acquired scientific and professional skills, increased their confidence and thinking like a scientist, cultivated a sense of science identity and developed interest in graduate school. Through the lens of social constructivism, we show how students’ immersion within the RECCS environment fostered a sense of belonging to the community of scientists’ students work with throughout the summer. The symbiotic relationship between mentors, mentees, and program staff is an example of the principles of Vygotsky’s "Zone of Proximal Development," as students are guided towards becoming confident and self-directed learners [34]. In the context of the Braided River career path framework [4], the RECCS program emerges as a potent tributary for community college students to pursue a STEM career. By aligning with Vygotsky’s principles, we recognize that RECCS students are active participants in the scientific community.

## Conclusion

Community colleges are an important component of the college landscape; about 54% of the U.S. population has attended community college at some point. Our data shows that early research opportunities for community college students such as RECCS can inspire students to advance a career in STEM. The supportive cohort and strong scaffolding appeared to be important success factors for the program. The personal growth towards becoming a STEM researcher that the students and their mentors described included a variety of research and communication skills, the development of a science identity and sense of belonging, and a vision for a career path within STEM. While RECCS community college students entered the program with different levels of preparation, mentors described them as hard-working and as achieving above-average quality in their research products, launching them into a STEM career path early in their academic career. RECCS students enter STEM careers at levels that well exceed the national average. This research clearly supports the value of continuing to invest in community college research opportunities. It not only demonstrates that community college students have both the capacity and work ethic to thrive in a research environment, but that the impact of these programs can help to shape career trajectories and increase persistence in STEM.

## Supporting information

S1 File Survey questions. (DOCX)

S2 File Survey data. (DOCX)

S3 File Cohen’s kappa for inter-rater reliability. (DOCX)
