# Supplementary material for: Early access to science research opportunities: Growth within a geoscience summer research program for community college students
Source: PLoS One. 2023 Dec 21;18(12):e0293674. doi: 10.1371/journal.pone.0293674 (PMC10734936; doi:10.1371/journal.pone.0293674)
Supplement: S1 File — (DOCX) [file pone.0293674.s001.docx]

Supporting Information (S1) for

Early access to science research opportunities: Growth within a geoscience summer research program for community college students

Christine Okochi^1 *^, Anne U. Gold^1^, Alicia Christensen^1^, Rebecca L. Batchelor^1^

^1^ Cooperative Institute for Research in Environmental Science, University of Colorado Boulder, Boulder, Colorado, United States of America

*Corresponding author

[christine.okochi@colorado.edu](mailto:christine.okochi@colorado.edu)

#

# **Survey questions.**

## **Student survey URSSA questions.**

1. **Thinking and Working like a Scientist**


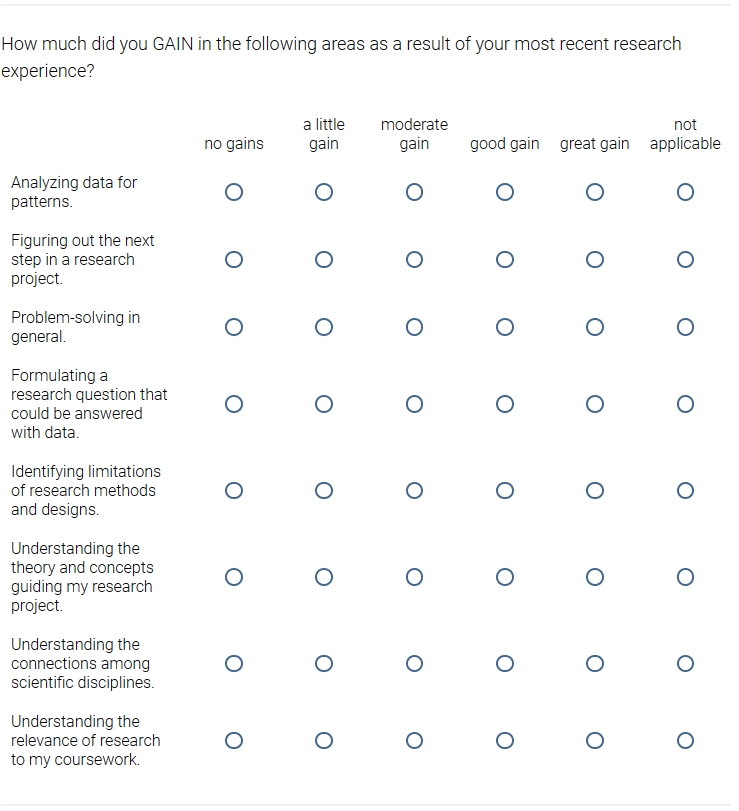


URSSA, 2009

1. **Personal and Professional Gains Related to Research**


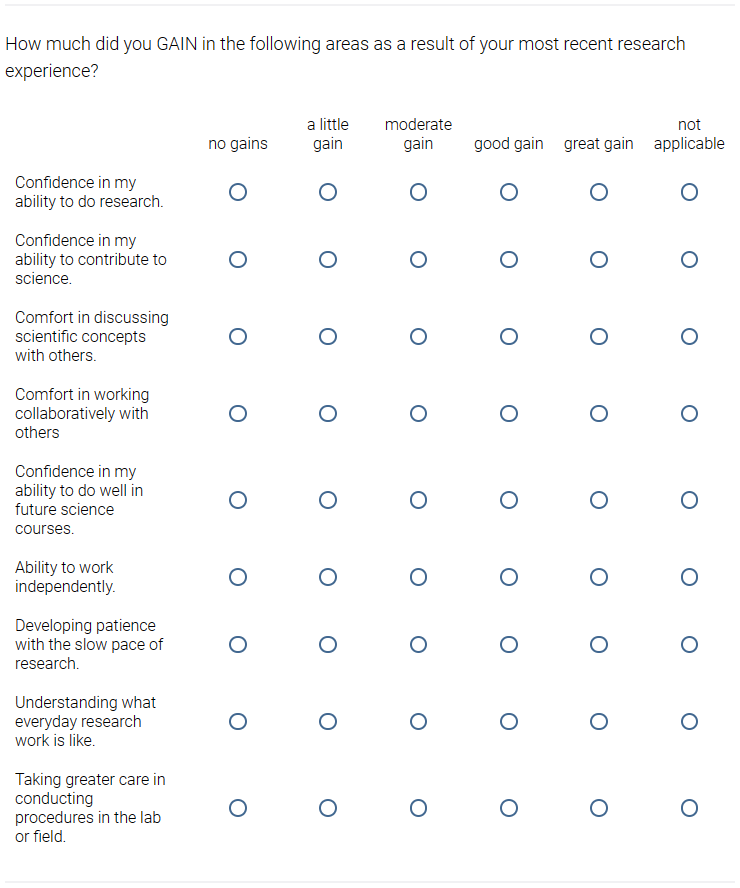


URSSA, 2009

1. **Gains in Research Skills**


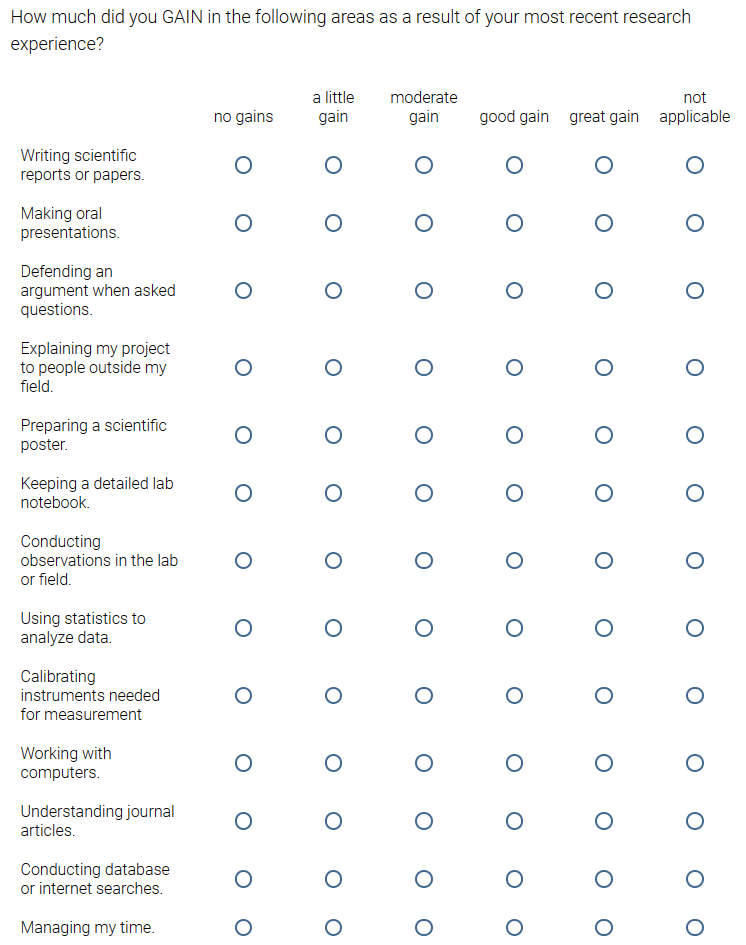


URSSA, 2009

1. **Engage in Attitudes and Behavior of a Researcher**


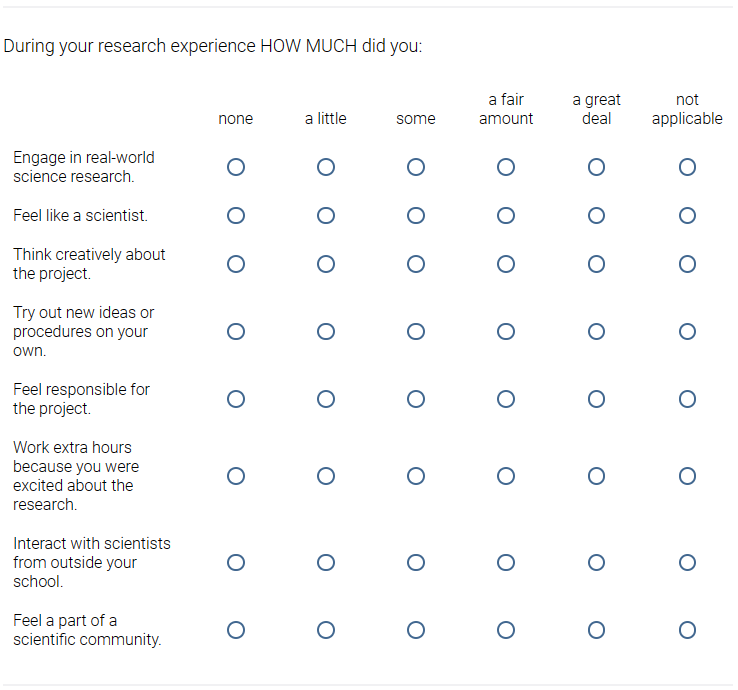


URSSA, 2009

1. **Impact of Research Experience on Future**

**
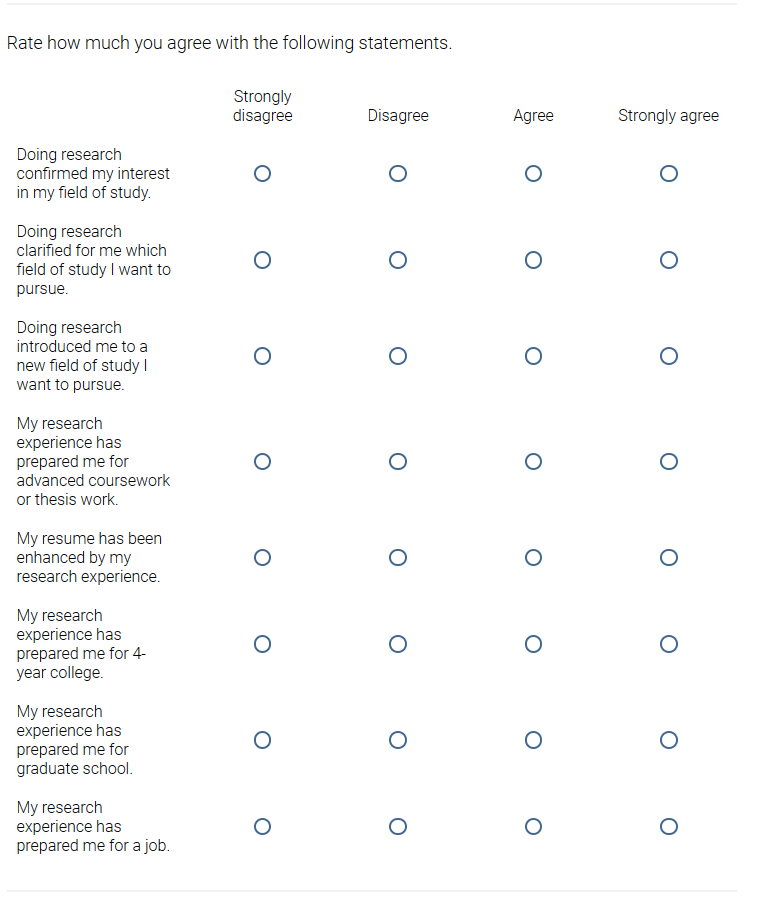
**

URSSA, 2009

1. **Impact of Research Experience on Future: Open-ended follow-up**


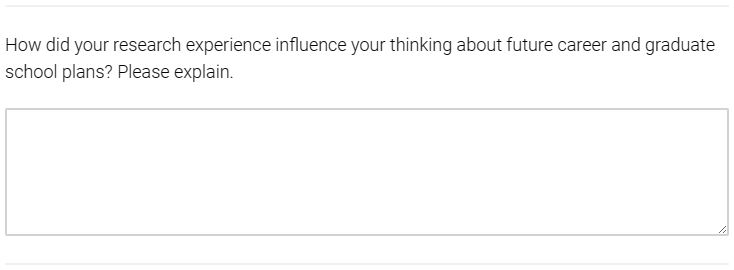


## **Mentor survey questions.**

1. **Mentor rating of student researcher**


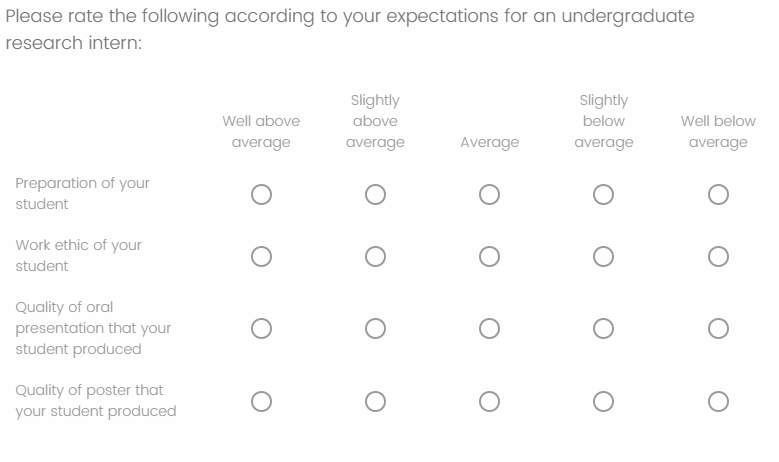


1. **Mentor rating of student progress as scientist-in-training**


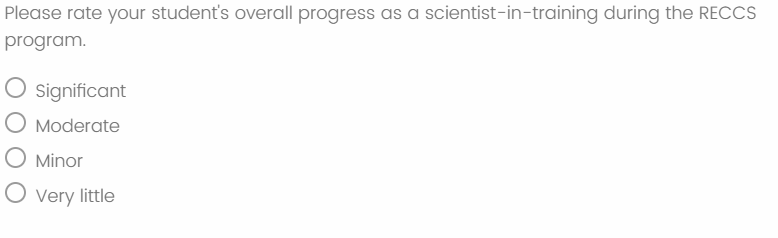


1. **Areas of most progress: Open-ended response**


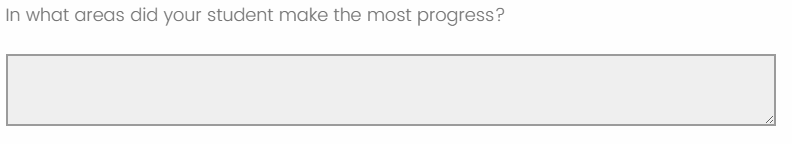


# **Reference**

URSSA. Undergraduate Research Student Self-Assessment. Ethnography & Evaluation Research, University of Colorado Boulder. 2009. Available from: [www.salgsite.org](http://www.salgsite.org)
