# Supplementary material for: Early access to science research opportunities: Growth within a geoscience summer research program for community college students
Source: PLoS One. 2023 Dec 21;18(12):e0293674. doi: 10.1371/journal.pone.0293674 (PMC10734936; doi:10.1371/journal.pone.0293674)
Supplement: S2 File — (DOCX) [file pone.0293674.s002.docx]

Supporting Information (S2) for

Early access to science research opportunities: Growth within a geoscience summer research program for community college students

Christine Okochi^1 *^, Anne U. Gold^1^, Alicia Christensen^1^, Rebecca L. Batchelor^1^

^1^ Cooperative Institute for Research in Environmental Science, University of Colorado Boulder, Boulder, Colorado, United States of America

*Corresponding author

[christine.okochi@colorado.edu](mailto:christine.okochi@colorado.edu)

# **Survey data.**

# Table 1: Self-report data for URSSA question *Thinking and Working Like a Scientist* (N=54).

Responses on a 5-point scale from 1= no gains to 5=great gains. No data = not applicable.

| Student ID | Analyzing data for patterns | Figuring out the next step in a research project | Problem-solving in general. | Formulating a research question that could be answered with data. | Identifying limitations of research methods and designs. | Understanding the theory and concepts guiding my research project. | Understanding the connections among scientific disciplines. | Understanding the relevance of research to my coursework. |
| --- | --- | --- | --- | --- | --- | --- | --- | --- |
| 1 | 5 | 4 | 5 | 4 | 5 | 4 | 5 | 5 |
| 2 | 4 | 4 | 4 | 4 | 4 | 4 | 5 | 5 |
| 3 | 4 | 4 | 4 | 4 | 4 | 3 | 3 | 4 |
| 4 | 4 | 5 | 4 | 5 | 5 | 5 | 5 | 5 |
| 5 | 4 | 4 | 3 | 1 | 4 | 4 | 4 | 4 |
| 6 | 5 | 5 | 5 | 5 | 5 | 5 | 5 | 5 |
| 7 | 5 | 5 | 5 | 5 | 5 | 5 | 5 | 5 |
| 8 | 5 | 4 | 5 | 5 | 5 | 4 | 5 | 5 |
| 9 | 3 | 4 | 5 | 3 | 5 | 5 | 3 | 3 |
| 10 | 4 | 4 | 5 | 5 | 5 | 3 | 5 | 4 |
| 11 | 5 | 5 | 4 | 5 | 5 | 5 | 5 | 4 |
| 12 | 5 | 3 | 5 | 4 | 4 | 5 | 3 | 5 |
| 13 | 2 | 3 | 4 | 5 | 5 | 3 | 4 | 2 |
| 14 | 5 | 5 |  |  | 5 | 4 | 4 | 5 |
| 15 |  |  |  | 4 | 5 |  | 3 | 1 |
| 16 | 5 | 5 | 5 | 5 | 5 | 4 | 3 | 4 |
| 17 | 5 | 5 | 5 | 5 | 4 | 5 | 4 | 5 |
| 18 | 5 | 5 | 5 | 5 | 4 | 5 | 5 | 4 |
| 19 | 5 | 5 | 5 | 5 | 5 | 5 | 4 | 5 |
| 20 | 5 | 4 | 5 | 3 | 5 | 5 | 4 | 5 |
| 21 | 5 | 5 | 5 | 5 | 3 | 5 | 5 | 5 |
| 22 | 4 | 3 | 3 | 3 | 3 | 4 | 4 | 5 |
| 23 | 5 | 5 | 5 | 5 | 5 | 5 | 5 | 5 |
| 24 | 5 | 5 | 5 | 5 | 5 | 5 | 5 | 5 |
| 25 | 4 | 4 | 4 | 4 | 3 | 4 | 4 | 4 |
| 26 | 5 | 5 | 5 | 5 | 5 | 5 | 5 | 5 |
| 27 | 5 | 5 | 5 | 5 | 5 | 5 | 5 | 5 |
| 28 | 5 | 5 | 4 | 5 | 4 | 5 | 5 | 5 |
| 29 | 5 | 5 | 5 | 5 | 5 | 5 | 5 | 5 |
| 30 | 5 | 5 | 4 | 4 | 5 | 4 | 5 | 3 |
| 31 | 5 | 5 | 5 | 5 | 5 | 5 | 5 | 5 |
| 32 | 3 | 5 | 2 | 5 | 4 | 5 | 4 | 4 |
| 33 | 4 | 5 | 4 | 4 | 3 | 5 | 5 | 5 |
| 34 | 5 | 5 | 5 | 5 | 5 | 5 | 5 | 5 |
| 35 | 3 | 3 | 4 | 4 | 4 | 4 | 4 | 4 |
| 36 | 5 | 5 | 5 | 5 | 5 | 5 | 2 | 5 |
| 37 |  | 3 | 3 |  | 5 | 5 | 5 | 3 |
| 38 | 4 | 4 | 4 | 4 | 4 | 4 | 4 | 4 |
| 39 | 5 | 5 | 5 | 5 | 5 | 5 | 5 | 5 |
| 40 | 5 | 5 | 4 | 4 | 5 | 5 | 5 | 4 |
| 41 | 1 | 3 | 2 | 4 | 5 | 4 | 3 | 4 |
| 42 | 5 | 5 | 5 | 4 | 4 | 4 | 3 | 3 |
| 43 | 5 | 5 | 4 | 4 | 5 | 5 | 5 | 5 |
| 44 | 4 | 4 | 5 | 4 | 5 | 4 | 4 | 4 |
| 45 | 4 | 5 | 5 | 4 | 5 | 3 | 4 |  |
| 46 | 5 | 5 | 5 | 5 | 5 | 5 | 5 | 5 |
| 47 | 5 | 5 | 5 | 5 | 5 | 5 | 5 | 5 |
| 48 | 5 | 5 | 5 | 5 | 5 | 5 | 5 | 5 |
| 49 | 5 | 5 | 5 | 5 | 5 | 5 | 5 | 5 |
| 50 | 5 | 5 | 5 | 5 | 5 | 5 | 5 | 5 |
| 51 | 3 | 4 | 4 |  | 5 |  |  |  |
| 52 | 5 | 5 | 5 | 5 | 5 | 5 | 5 | 5 |
| 53 | 5 | 5 | 5 | 5 | 5 | 5 | 5 | 5 |
| 54 | 5 | 4 | 4 | 3 | 5 | 5 | 3 | 4 |

# Table 2: Self-report data for URSSA question *Personal and Professional Gains related to Research* (N=54).

Responses on a 5-point scale from 1= no gains to 5=great gains. No data = not applicable.

| Student ID | Confidence in my ability to do research. | Confidence in my ability to contribute to science. | Comfort in discussing scientific concepts with others. | Comfort in working collaboratively with others. | Confidence in my ability to do well in future science courses. | Ability to work independently | Developing patience with the slow pace of research. | Understanding what everyday research work is like. | Taking greater care in conducting procedures in the lab or field. |
| --- | --- | --- | --- | --- | --- | --- | --- | --- | --- |
| 1 | 4 | 5 | 4 | 5 | 5 | 4 | 5 | 5 | 5 |
| 2 | 5 | 5 | 5 | 4 | 5 | 4 | 4 | 5 | 4 |
| 3 | 4 | 4 | 4 | 4 | 4 | 4 | 4 | 4 | 4 |
| 4 | 5 | 5 | 4 | 5 | 5 | 5 | 5 | 5 | 5 |
| 5 | 5 | 5 | 5 | 5 | 5 | 1 | 5 | 5 | 1 |
| 6 | 5 | 5 | 5 | 5 | 5 | 5 | 5 | 5 | 5 |
| 7 | 5 | 5 | 5 | 5 | 5 | 5 |  | 5 |  |
| 8 | 5 | 5 | 5 | 5 | 5 | 5 | 5 | 4 | 5 |
| 9 | 3 | 3 | 3 | 5 | 3 | 4 | 5 | 5 | 5 |
| 10 | 4 | 4 | 4 | 4 | 4 | 5 | 5 | 5 | 5 |
| 11 | 5 | 5 | 5 | 5 | 5 | 4 | 4 | 5 | 5 |
| 12 | 5 | 5 | 5 | 5 | 4 | 5 | 5 | 5 | 5 |
| 13 | 5 | 5 | 4 | 5 | 5 | 5 | 4 | 4 | 5 |
| 14 | 5 | 4 | 3 | 4 | 4 | 5 | 4 | 5 | 4 |
| 15 | 5 | 5 | 5 | 5 | 5 | 3 | 5 | 5 | 5 |
| 16 | 5 | 5 | 4 | 4 | 5 | 5 | 3 | 4 | 4 |
| 17 | 5 | 5 | 5 | 5 | 4 | 4 | 3 | 5 | 5 |
| 18 | 5 | 5 | 4 | 4 | 4 | 5 | 5 | 5 | 5 |
| 19 | 5 | 5 | 5 | 5 | 5 | 4 | 4 | 5 | 5 |
| 20 | 5 | 4 | 5 | 4 | 5 | 4 | 5 | 5 | 5 |
| 21 | 5 | 5 | 4 | 4 | 5 | 3 | 4 | 5 | 5 |
| 22 | 4 | 4 | 4 | 5 | 5 | 5 | 4 | 5 | 5 |
| 23 | 5 | 5 | 5 | 5 | 5 | 5 | 5 | 5 | 5 |
| 24 | 5 | 5 | 5 | 5 | 5 | 5 | 4 | 5 | 5 |
| 25 | 4 | 4 | 4 | 4 | 5 | 4 |  | 3 | 3 |
| 26 | 5 | 5 | 5 | 5 | 5 | 5 | 5 | 5 | 5 |
| 27 | 5 | 5 | 5 | 5 | 5 | 4 | 4 | 5 | 5 |
| 28 | 5 | 5 | 5 | 5 | 5 | 5 | 5 | 5 | 4 |
| 29 | 5 | 5 | 5 | 5 | 5 | 5 | 5 | 5 | 5 |
| 30 | 5 | 5 | 5 | 5 | 5 | 4 | 3 | 5 |  |
| 31 | 5 | 5 | 4 | 5 | 5 | 5 | 5 | 5 |  |
| 32 | 5 | 5 | 4 | 5 | 4 | 4 | 4 | 5 | 5 |
| 33 | 4 | 5 | 5 | 4 | 4 | 5 | 5 | 3 | 2 |
| 34 | 5 | 5 | 5 | 5 | 5 | 5 | 5 | 5 | 5 |
| 35 | 4 | 4 | 4 | 4 | 4 | 4 | 4 | 4 | 4 |
| 36 | 5 | 5 | 5 | 5 | 1 | 2 | 4 | 5 | 4 |
| 37 | 3 | 3 | 3 | 5 | 3 | 3 | 4 | 4 |  |
| 38 | 4 | 4 | 4 | 4 | 4 | 4 | 4 | 4 | 4 |
| 39 | 5 | 5 | 4 | 5 | 4 | 5 | 5 | 5 | 5 |
| 40 | 5 | 5 | 5 | 5 | 4 | 4 | 4 | 4 | 4 |
| 41 | 5 | 5 | 3 | 4 | 5 | 2 | 3 | 5 | 2 |
| 42 | 5 | 5 | 4 | 4 | 5 |  | 4 | 5 | 5 |
| 43 | 5 | 5 | 5 | 5 | 5 | 5 | 5 | 5 | 4 |
| 44 | 5 | 5 | 4 | 4 | 5 | 5 | 4 | 4 | 4 |
| 45 | 5 | 5 | 5 | 3 | 5 | 5 | 5 | 5 | 4 |
| 46 | 5 | 5 | 3 | 3 | 5 | 5 | 5 | 5 | 4 |
| 47 | 5 | 5 | 5 | 5 | 5 | 5 | 5 | 5 | 5 |
| 48 | 4 | 5 | 5 | 2 | 5 | 3 | 5 | 4 | 4 |
| 49 | 5 | 5 | 5 | 5 | 5 | 5 | 4 | 5 | 5 |
| 50 | 5 | 5 | 5 | 5 | 5 | 5 | 5 | 5 | 5 |
| 51 | 5 | 5 |  | 4 |  |  |  |  | 5 |
| 52 | 5 | 5 | 5 | 5 | 5 | 5 | 5 | 5 | 5 |
| 53 | 5 | 5 | 5 | 5 | 5 | 5 | 5 | 5 | 4 |
| 54 | 5 | 5 | 5 | 4 | 5 | 4 | 2 | 5 | 3 |

# Table 3: Self-report data for URSSA question *Gains in Research Skills* (N=54).

Responses on a 5-point scale from 1= no gains to 5=great gains. No data = not applicable.

| Student ID | Writing scientific reports or papers. | Making oral presentations. | Defending an argument when asked questions. | Explaining my project to people outside my field. | Preparing a scientific poster. | Keeping a detailed lab notebook. | Conducting observations in the lab or field. | Using statistics to analyze data. | Calibrating instruments needed for measurement. | Working with computers. | Understanding journal articles. | Conducting database or internet searches. | Managing my time. |
| --- | --- | --- | --- | --- | --- | --- | --- | --- | --- | --- | --- | --- | --- |
| 1 | 4 | 4 | 5 | 5 | 5 | 4 | 2 | 5 | 1 | 5 | 3 | 4 | 4 |
| 2 | 4 | 4 | 4 | 4 | 4 | 4 | 4 | 3 | 4 | 4 | 4 | 4 | 4 |
| 3 | 4 | 4 | 4 | 4 | 4 | 3 | 2 | 4 | 4 | 4 | 4 | 4 | 4 |
| 4 | 5 | 5 | 4 | 4 | 5 | 5 | 5 | 5 | 4 | 5 | 4 | 5 | 5 |
| 5 | 4 | 5 | 1 | 5 | 5 | 1 | 3 | 5 | 1 | 5 | 5 | 5 | 5 |
| 6 | 5 | 5 | 5 | 5 | 5 | 5 | 5 | 5 | 5 | 5 | 5 | 5 | 5 |
| 7 | 5 | 5 | 5 | 5 | 5 | 4 | 5 | 5 |  | 5 | 5 | 5 | 5 |
| 8 | 4 | 5 | 4 | 5 | 5 | 4 | 5 | 4 |  | 4 | 4 | 1 | 5 |
| 9 | 4 | 4 | 3 | 4 | 4 | 4 | 4 | 3 | 3 | 3 | 4 | 4 | 5 |
| 10 | 5 | 5 | 3 | 4 | 5 | 5 | 5 | 3 |  | 4 | 4 | 4 | 3 |
| 11 | 4 | 4 | 5 | 5 | 5 | 5 | 5 | 5 |  | 4 | 4 | 4 | 4 |
| 12 | 3 | 5 | 3 | 5 | 5 | 5 | 5 | 4 |  | 5 | 3 | 5 | 5 |
| 13 | 2 | 4 | 4 | 5 | 5 | 2 | 4 | 4 | 5 | 2 | 3 | 2 | 5 |
| 14 | 4 | 5 | 4 | 5 | 5 | 2 | 3 | 5 | 3 | 5 | 4 | 4 | 5 |
| 15 | 5 | 5 | 5 | 5 | 5 | 5 | 5 | 5 | 1 | 5 | 3 | 5 | 5 |
| 16 | 5 | 4 | 4 | 5 | 5 | 5 | 5 | 5 |  | 4 | 4 | 4 | 3 |
| 17 | 5 | 4 | 4 | 3 | 5 | 5 | 5 | 4 | 4 | 4 | 4 | 3 | 4 |
| 18 | 5 | 4 | 4 | 4 | 5 | 3 | 4 | 5 | 4 | 5 | 4 | 4 | 4 |
| 19 | 5 | 5 | 4 | 5 | 5 | 5 | 5 | 5 | 3 | 5 | 5 | 3 | 5 |
| 20 | 4 | 5 | 4 | 5 | 5 | 3 | 4 | 3 | 4 | 4 | 4 | 3 | 4 |
| 21 | 5 | 5 | 5 | 5 | 5 | 2 | 4 | 4 |  | 5 | 5 | 5 | 5 |
| 22 | 4 | 3 | 3 | 4 | 5 | 5 | 5 | 3 | 4 | 3 | 3 | 3 | 4 |
| 23 | 5 | 5 | 5 | 5 | 5 | 5 | 5 | 5 | 5 | 5 | 5 | 5 | 5 |
| 24 | 4 | 5 | 5 | 5 | 5 | 4 | 5 | 4 | 5 | 5 | 5 | 5 | 5 |
| 25 | 3 | 3 | 3 | 4 | 4 |  |  | 5 | 4 | 5 | 4 | 5 | 3 |
| 26 | 5 | 5 | 5 | 5 | 5 | 5 | 5 | 5 | 5 | 5 | 5 | 5 | 5 |
| 27 | 5 | 5 | 5 | 5 | 5 | 5 | 5 | 5 | 5 | 5 | 5 | 5 | 4 |
| 28 | 4 | 5 | 5 | 5 | 5 | 4 | 3 | 5 | 2 | 5 | 4 |  | 5 |
| 29 | 4 | 4 | 3 | 4 | 5 | 3 | 3 | 4 | 5 | 5 | 3 | 3 | 3 |
| 30 | 5 | 4 | 4 | 5 | 5 | 5 |  | 5 |  | 5 | 3 | 3 | 3 |
| 31 | 4 | 5 | 5 | 5 | 5 | 5 |  | 5 |  | 4 | 5 | 5 | 5 |
| 32 | 4 | 4 | 4 | 5 | 5 | 3 | 4 | 2 | 2 | 5 | 4 | 5 | 4 |
| 33 | 2 | 4 | 5 | 5 | 5 | 4 | 2 | 5 | 4 | 5 | 3 | 3 | 4 |
| 34 | 5 | 5 | 5 | 5 | 5 | 5 | 4 |  | 5 | 5 | 5 | 5 |  |
| 35 | 4 | 4 | 4 | 5 | 4 | 4 | 4 | 3 | 5 | 4 | 4 | 4 | 4 |
| 36 | 4 | 4 | 4 | 5 | 3 |  | 4 | 5 |  | 5 | 5 | 4 | 1 |
| 37 | 5 | 4 | 4 | 5 | 5 |  |  | 5 |  | 3 | 3 |  | 3 |
| 38 |  | 5 | 4 | 4 | 4 |  | 4 | 4 |  | 4 | 4 | 4 | 4 |
| 39 | 5 | 5 | 5 | 5 | 5 | 4 | 4 | 4 | 5 | 5 | 5 | 5 | 5 |
| 40 | 5 | 5 | 4 | 5 | 5 | 3 | 3 | 5 |  | 5 | 5 | 4 | 4 |
| 41 | 4 | 3 | 3 | 3 | 5 | 3 | 3 | 1 | 2 | 2 | 4 | 2 | 3 |
| 42 | 4 | 4 | 4 | 5 | 4 | 4 | 5 | 4 | 2 | 3 | 5 | 5 | 5 |
| 43 | 5 | 4 | 3 | 4 | 5 | 4 | 4 | 5 |  | 5 | 4 | 4 | 4 |
| 44 | 4 | 4 | 3 | 5 | 4 | 5 | 5 |  |  | 3 | 4 | 5 | 5 |
| 45 | 3 | 4 | 4 | 5 | 4 | 5 | 5 | 4 | 5 | 4 | 4 | 3 | 4 |
| 46 | 4 | 4 | 4 | 5 | 4 | 4 | 4 | 5 | 5 | 4 | 5 | 5 | 5 |
| 47 | 5 | 5 | 5 | 5 | 5 | 5 | 5 | 5 | 5 | 5 | 5 | 5 | 5 |
| 48 |  | 5 | 5 | 5 | 5 | 3 | 4 |  |  | 5 | 4 | 3 | 4 |
| 49 | 5 | 5 | 4 | 5 | 5 | 4 | 5 | 4 | 5 | 3 | 4 | 4 | 5 |
| 50 |  | 5 | 5 | 5 | 5 |  |  | 5 |  | 5 | 5 | 5 | 5 |
| 51 | 4 |  | 5 |  |  | 5 | 4 | 4 | 2 | 2 | 5 | 5 | 4 |
| 52 | 4 | 5 | 5 | 5 | 5 | 5 | 5 | 5 | 4 | 5 | 5 | 4 | 5 |
| 53 | 4 | 5 | 5 | 5 | 5 | 5 | 4 | 5 | 4 | 5 | 5 | 5 | 5 |
| 54 | 2 | 4 | 3 | 5 | 5 | 3 | 4 | 5 | 5 | 5 | 5 | 4 | 4 |

# Table 4: Self-report data for URSSA question *Engaging in Attitudes and Behaviors of a Researcher* (N=54).

Responses on a 5-point scale from 1= none to 5=a great deal. No data = not applicable.

| Student ID | Engage in real-world science research | Feel like a scientist. | Think creatively about the project. | Try out new ideas or procedures on your own. | Feel responsible for the project. | Work extra hours because you were excited about the research. | Interact with scientists from outside your school. | Feel a part of a scientific community. |
| --- | --- | --- | --- | --- | --- | --- | --- | --- |
| 1 | 5 | 4 | 5 | 3 | 5 | 3 | 5 | 5 |
| 2 | 4 | 4 | 5 | 4 | 4 | 5 | 5 | 4 |
| 3 | 5 | 5 | 5 | 5 | 5 | 5 | 5 | 5 |
| 4 | 5 | 5 | 5 | 5 | 5 | 5 | 5 | 5 |
| 5 | 4 | 5 | 5 | 4 | 5 | 5 | 5 | 5 |
| 6 | 5 | 5 | 5 | 5 | 5 | 5 | 5 | 5 |
| 7 | 5 | 5 | 5 | 5 | 5 | 5 | 5 | 5 |
| 8 | 5 | 5 | 4 | 3 | 4 | 3 | 5 | 5 |
| 9 | 4 | 5 | 5 | 4 | 5 | 5 | 2 | 3 |
| 10 | 5 | 4 | 4 | 2 | 5 | 3 | 4 | 3 |
| 11 | 5 | 4 | 5 | 5 | 5 | 4 | 5 | 4 |
| 12 | 5 | 5 | 4 | 5 | 5 | 4 | 5 | 5 |
| 13 | 4 | 5 | 5 | 4 | 5 | 4 | 2 | 5 |
| 14 | 5 | 5 | 5 | 5 | 5 | 5 | 5 | 5 |
| 15 | 5 | 5 | 5 | 5 | 5 | 5 | 5 | 5 |
| 16 | 5 | 5 | 5 | 5 | 5 | 5 | 4 | 4 |
| 17 | 5 | 5 | 4 |  | 5 | 5 | 5 | 5 |
| 18 | 5 | 5 | 5 | 3 | 5 | 4 | 3 | 4 |
| 19 | 5 | 5 | 5 | 4 | 5 | 4 | 5 | 5 |
| 20 | 5 | 4 | 3 | 4 | 3 | 4 | 3 | 4 |
| 21 | 5 | 5 | 4 | 4 | 5 | 5 | 3 | 5 |
| 22 | 5 | 5 | 5 | 5 | 5 | 5 | 5 | 5 |
| 23 | 5 | 5 | 5 | 5 | 5 | 5 | 5 | 5 |
| 24 | 5 | 5 | 5 | 5 | 5 | 5 | 5 | 5 |
| 25 | 5 | 4 | 5 | 5 | 5 | 3 | 5 | 5 |
| 26 | 5 | 4 | 5 | 5 | 5 | 5 | 5 | 5 |
| 27 | 5 | 5 | 5 | 5 | 5 | 4 | 5 | 5 |
| 28 | 5 | 5 | 5 | 5 | 5 | 5 | 5 | 5 |
| 29 | 5 | 4 | 4 | 4 | 4 | 4 | 5 | 4 |
| 30 | 5 | 5 | 5 | 5 | 5 | 4 | 5 | 5 |
| 31 | 5 | 5 | 5 | 5 | 5 | 5 | 5 | 5 |
| 32 | 5 | 4 | 4 | 3 | 5 | 2 | 2 | 2 |
| 33 | 3 | 4 | 5 | 4 | 5 | 3 | 4 | 5 |
| 34 | 5 | 5 | 3 | 3 | 5 | 5 | 5 | 5 |
| 35 | 5 | 5 | 5 | 5 | 5 | 5 |  | 5 |
| 36 | 5 | 5 | 5 | 5 | 5 | 4 | 5 | 5 |
| 37 | 3 | 2 | 1 | 1 | 4 | 2 | 4 | 3 |
| 38 | 5 | 3 | 3 | 4 | 4 | 4 | 3 | 4 |
| 39 | 5 | 5 | 5 | 4 | 5 | 5 | 5 | 5 |
| 40 | 5 | 5 | 5 | 4 | 5 | 4 | 5 | 5 |
| 41 | 3 | 5 | 5 | 3 | 4 | 3 | 3 | 5 |
| 42 | 4 | 4 | 4 | 4 | 4 | 5 | 5 | 5 |
| 43 | 4 | 4 | 5 | 5 | 5 | 5 | 3 | 4 |
| 44 | 4 | 4 | 5 | 5 | 5 | 5 | 5 | 5 |
| 45 | 5 | 4 | 4 | 4 | 5 | 3 | 3 | 2 |
| 46 | 4 | 5 | 5 | 5 | 3 | 4 | 4 | 5 |
| 47 | 5 | 5 | 5 | 5 | 5 | 5 | 5 | 5 |
| 48 | 5 | 5 | 5 | 5 | 5 | 4 | 5 | 5 |
| 49 | 5 | 4 | 4 | 3 | 5 | 5 | 5 | 5 |
| 50 | 5 | 5 | 5 | 5 | 5 | 5 | 5 | 5 |
| 51 | 5 | 5 | 5 | 4 | 5 | 5 | 4 | 5 |
| 52 | 5 | 5 | 5 | 5 | 5 | 5 | 5 | 5 |
| 53 | 5 | 5 | 5 | 5 | 5 | 5 | 4 | 5 |
| 54 | 5 | 5 | 5 | 2 | 4 | 5 | 5 | 5 |

# Table 5: Self-report data for URSSA question *Impact of Research Experience* (N=54).

Responses on a 4-point scale from 1= strongly disagree to 5=strongly agree.

| Student ID | Doing research confirmed my interest in my field of study. | Doing research clarified for me which field of study I want to pursue. | Doing research introduced me to a new field of study I want to pursue. | My research experience has prepared me for advanced coursework or thesis work. | My resume has been enhanced by my research experience. | My research experience has prepared me for 4-year college. | My research experience has prepared me for graduate school. | My research experience has prepared me for a job. |
| --- | --- | --- | --- | --- | --- | --- | --- | --- |
| 1 | 3 | 3 | 2 | 4 | 4 | 4 | 4 | 4 |
| 2 | 3 | 3 | 3 | 3 | 3 | 3 | 3 | 3 |
| 3 | 4 | 4 | 3 | 4 | 4 | 4 | 4 | 4 |
| 4 | 4 | 3 | 4 | 4 | 4 | 4 | 3 | 4 |
| 5 | 3 | 2 | 3 | 3 | 3 | 3 | 3 | 4 |
| 6 | 4 | 4 | 4 | 3 | 4 | 4 | 4 | 4 |
| 7 | 4 | 3 | 3 | 4 | 4 | 4 | 4 | 4 |
| 8 | 4 | 4 | 2 | 4 | 4 | 4 | 4 | 3 |
| 9 | 3 | 3 | 3 | 3 | 3 | 3 | 2 | 3 |
| 10 | 4 | 3 | 2 | 3 | 4 | 3 | 2 | 2 |
| 11 | 4 | 3 | 3 | 4 | 4 | 4 | 3 | 3 |
| 12 | 4 | 3 | 3 | 3 | 4 | 3 | 3 | 4 |
| 13 | 3 | 4 | 1 | 3 | 4 | 4 | 4 | 4 |
| 14 | 3 | 3 | 3 | 3 | 3 | 4 | 4 | 4 |
| 15 | 2 | 3 | 3 | 4 | 4 | 4 | 4 | 4 |
| 16 | 3 | 4 | 4 | 2 | 3 | 4 | 1 | 2 |
| 17 | 4 | 4 | 3 | 4 | 4 | 4 | 4 | 4 |
| 18 | 4 | 2 | 2 | 3 | 4 | 4 | 3 | 3 |
| 19 | 4 | 4 | 4 | 4 | 4 | 4 | 4 | 4 |
| 20 | 3 | 2 | 2 | 4 | 4 | 4 | 4 | 3 |
| 21 | 4 | 4 | 3 | 4 | 4 | 4 | 4 | 4 |
| 22 | 4 | 2 | 4 | 4 | 4 | 4 | 4 | 4 |
| 23 | 4 | 4 | 4 | 4 | 4 | 4 | 4 | 4 |
| 24 | 4 | 3 | 2 | 4 | 4 | 4 | 4 | 4 |
| 25 | 4 | 2 | 2 | 3 | 3 | 3 | 3 | 3 |
| 26 | 4 | 4 | 3 | 4 | 4 | 4 | 4 | 4 |
| 27 | 4 | 3 | 2 | 4 | 4 | 4 | 4 | 4 |
| 28 | 3 | 3 | 3 | 4 | 4 | 4 | 4 | 4 |
| 29 | 3 | 3 | 2 | 4 | 4 | 3 | 3 | 3 |
| 30 | 3 | 4 | 2 | 4 | 4 | 4 | 4 | 3 |
| 31 | 3 | 4 | 3 | 4 | 4 | 4 | 4 | 4 |
| 32 | 2 | 2 | 2 | 3 | 4 | 3 | 3 | 3 |
| 33 | 3 | 3 | 4 | 3 | 3 | 4 | 3 | 4 |
| 34 | 3 | 3 | 3 | 3 | 4 | 3 | 3 | 3 |
| 35 | 2 | 2 | 3 | 3 | 3 | 3 | 3 | 3 |
| 36 | 4 | 4 | 2 | 4 | 4 | 4 | 4 | 4 |
| 37 | 1 | 3 | 1 | 3 | 3 | 3 | 2 | 1 |
| 38 | 3 | 3 | 1 | 4 | 4 | 4 | 4 | 3 |
| 39 | 3 | 3 | 2 | 3 | 3 | 3 | 3 | 3 |
| 40 | 2 | 2 | 2 | 3 | 4 | 3 | 3 | 3 |
| 41 | 4 | 4 | 4 | 4 | 4 | 3 | 4 | 3 |
| 42 | 3 | 2 | 2 | 2 | 3 | 3 | 2 | 3 |
| 43 | 4 | 3 | 3 | 4 | 4 | 4 | 4 | 3 |
| 44 | 3 | 2 | 3 | 4 | 4 | 4 | 3 | 4 |
| 45 | 4 | 2 | 2 | 3 | 4 | 3 | 3 | 4 |
| 46 | 3 | 3 | 2 | 4 | 4 | 2 | 3 | 3 |
| 47 | 4 | 4 | 4 | 4 | 4 | 4 | 4 | 4 |
| 48 | 4 | 4 | 4 | 4 | 4 | 4 | 4 | 3 |
| 49 | 4 | 4 | 3 | 3 | 4 | 4 | 3 | 4 |
| 50 | 4 | 4 | 2 | 2 | 4 | 4 | 4 | 4 |
| 51 | 3 | 3 | 1 | 3 | 4 | 4 | 4 | 4 |
| 52 | 4 | 4 | 2 | 4 | 4 | 4 | 4 | 4 |
| 53 | 3 | 3 | 3 | 4 | 4 | 4 | 4 | 4 |
| 54 | 1 | 4 | 4 | 3 | 4 | 4 | 4 | 4 |

# Table 6: Self-report data for each item in the URSSA question blocks for RECCS students from 2015-2019 (N=54).

Responses on a scale of 1 to 5; data reported as mean for each cohort and for the study group (all N=54 students).

|  | **2019 (n=13)** | **2018 (n=10)** | **2017 (n=11)** | **2016 (n=10)** | **2015 (n=10)** | **All (n=54)** |
| --- | --- | --- | --- | --- | --- | --- |
| **Gains in thinking and working like a scientist:** | **4.63** | **4.27** | **4.67** | **4.45** | **4.39** | **4.48** |
| Analyzing data for patterns | 4.69 | 3.89 | 4.82 | 4.67 | 4.30 | **4.47** |
| Figuring out the next step in a research project | 4.77 | 4.30 | 4.73 | 4.44 | 4.30 | **4.51** |
| Problem-solving in general. | 4.77 | 3.80 | 4.55 | 4.75 | 4.50 | **4.47** |
| Formulating a research question that could be answered with data. | 4.50 | 4.44 | 4.64 | 4.56 | 4.10 | **4.45** |
| Identifying limitations of research methods and designs. | 4.92 | 4.50 | 4.36 | 4.70 | 4.70 | **4.64** |
| Understanding the theory and concepts guiding my research project. | 4.25 | 4.70 | 4.73 | 4.56 | 4.20 | **4.49** |
| Understanding the connections among scientific disciplines. | 4.50 | 4.20 | 4.82 | 3.90 | 4.50 | **4.38** |
| Understanding the relevance of research to my coursework. | 4.64 | 4.30 | 4.73 | 4.00 | 4.50 | **4.43** |
| **Personal gains related to research work:** | **4.67** | **4.22** | **4.72** | **4.69** | **4.54** | **4.56** |
| Confidence in my ability to do research. | 4.92 | 4.50 | 4.80 | 5.00 | 4.50 | **4.74** |
| Confidence in my ability to contribute to science. | 5.00 | 4.60 | 4.82 | 4.80 | 4.60 | **4.76** |
| Comfort in discussing scientific concepts with others. | 4.67 | 4.20 | 4..6 | 4.50 | 4.40 | **4.44** |
| Comfort in working collaboratively with others | 4.15 | 4.60 | 4.80 | 4.60 | 4.70 | **4.57** |
| Confidence in my ability to do well in future science courses. | 5.00 | 3.80 | 5.00 | 4.60 | 4.60 | **4.60** |
| Ability to work independently | 4.73 | 3.80 | 4.55 | 4.40 | 4.20 | **4.33** |
| Developing patience with the slow pace of research. | 4.30 | 4.20 | 4.30 | 4.70 | 4.78 | **4.46** |
| Understanding what everyday research work is like. | 4.83 | 4.40 | 4.82 | 4.80 | 4.80 | **4.73** |
| Taking greater care in conducting procedures in the lab or field. | 4.38 | 3.89 | 4.70 | 4.80 | 4.30 | **4.41** |
| **Gains in research skills:** | **4.44** | **4.07** | **4.47** | **4.24** | **4.18** | **4.28** |
| Writing scientific reports or papers. | 4.00 | 4.22 | 4.36 | 4.20 | 4.40 | **4.24** |
| Making oral presentations. | 4.50 | 4.30 | 4.45 | 4.50 | 4.60 | **4.47** |
| Defending an argument when asked questions. | 4.23 | 4.20 | 4.36 | 4.10 | 3.80 | **4.14** |
| Explaining my project to people outside my field. | 4.92 | 4.70 | 4.73 | 4.70 | 4.50 | **4.71** |
| Preparing a scientific poster. | 4.67 | 4.60 | 4.91 | 5.00 | 4.70 | **4.78** |
| Keeping a detailed lab notebook. | 4.33 | 3.71 | 4.30 | 4.00 | 3.90 | **4.05** |
| Conducting observations in the lab or field. | 4.50 | 3.56 | 4.38 | 4.50 | 4.00 | **4.19** |
| Using statistics to analyze data. | 4.64 | 3.78 | 4.55 | 4.50 | 4.20 | **4.33** |
| Calibrating instruments needed for measurement | 4.11 | 3.83 | 4.38 | 3.43 | 3.10 | **3.77** |
| Working with computers. | 4.15 | 4.30 | 4.73 | 4.30 | 4.40 | **4.38** |
| Understanding journal articles. | 4.62 | 4.20 | 4.27 | 3.80 | 4.20 | **4.22** |
| Conducting database or internet searches. | 4.38 | 4.00 | 4.40 | 3.70 | 4.10 | **4.12** |
| Managing my time. | 4.62 | 3.56 | 4.27 | 4.40 | 4.50 | **4.27** |
| **Engage in the attitudes and behaviors of a researcher:** | **4.63** | **4.15** | **4.82** | **4.59** | **4.56** | **4.55** |
| Engage in real-world science research | 4.69 | 4.40 | 5.00 | 4.90 | 4.70 | **4.74** |
| Feel like a scientist. | 4.62 | 4.30 | 4.73 | 4.80 | 4.70 | **4.63** |
| Think creatively about the project. | 4.77 | 4.10 | 4.82 | 4.60 | 4.80 | **4.62** |
| Try out new ideas or procedures on your own. | 4.38 | 3.60 | 4.82 | 4.44 | 4.00 | **4.25** |
| Feel responsible for the project. | 4.69 | 4.70 | 4.91 | 4.80 | 4.80 | **4.78** |
| Work extra hours because you were excited about the research. | 4.69 | 3.70 | 4.55 | 4.40 | 4.40 | **4.35** |
| Interact with scientists from outside your school. | 4.46 | 4.00 | 4.82 | 4.20 | 4.60 | **4.42** |
| Feel a part of a scientific community. | 4.69 | 4.40 | 4.91 | 4.60 | 4.50 | **4.62** |

#

# Table 7: Mentor perceptions of student preparation, work ethic, and quality of deliverables in terms of their expectations for undergraduate researchers (n=48 students, rated by 63 mentors).

Responses on a 4-point scale from 1=well below average to 5=well above average. For students who were mentored by more than one person, an average of their mentors’ ratings is shown.

| **Student ID** | **Preparation of your student** | **Work ethic of your student** | **Quality of presentation** | **Quality of poster** |
| --- | --- | --- | --- | --- |
| 1 | 4.0 | 5.0 | 4.0 | 5.0 |
| 2 | 5.0 | 5.0 | 5.0 | 5.0 |
| 3 | 1.0 | 2.0 | 1.0 | 3.0 |
| 4 | 5.0 | 5.0 | 5.0 | 5.0 |
| 5 | 2.0 | 3.0 | 2.0 | 2.0 |
| 6 | 3.0 | 5.0 | 4.0 | 4.0 |
| 7 | 1.5 | 5.0 | 4.5 | 4.5 |
| 8 | 5.0 | 5.0 | 4.0 | 5.0 |
| 9 | 3.0 | 5.0 | 4.0 | 4.0 |
| 10 | 2.5 | 3.0 | 2.5 | 2.5 |
| 12 | 4.0 | 5.0 | 5.0 | 4.0 |
| 13 | 2.0 | 1.0 | 2.0 | 2.0 |
| 14 | 3.0 | 1.0 | 3.0 | 3.0 |
| 15 | 4.0 | 5.0 | 5.0 | 4.0 |
| 16 | 4.0 | 5.0 | 4.0 | 4.0 |
| 17 | 4.0 | 4.0 | 3.0 | 3.0 |
| 18 | 3.0 | 5.0 | 3.0 | 3.0 |
| 19 | 3.0 | 5.0 | 4.0 | 5.0 |
| 20 | 4.0 | 3.0 | 4.0 | 4.0 |
| 22 | 4.0 | 3.0 | 3.0 | 4.0 |
| 23 | 3.5 | 5.0 | 4.0 | 3.5 |
| 24 | 3.0 | 4.0 | 4.0 | 4.0 |
| 25 | 5.0 | 5.0 | 4.0 | 5.0 |
| 26 | 2.0 | 1.0 | 4.0 | 3.0 |
| 27 | 4.0 | 5.0 | 4.0 | 5.0 |
| 28 | 3.0 | 2.0 | 1.0 | 1.0 |
| 30 | 5.0 | 5.0 | 5.0 | 5.0 |
| 31 | 5.0 | 3.0 | 5.0 | 4.0 |
| 32 | 2.5 | 1.5 | 2.0 | 3.0 |
| 33 | 3.0 | 3.0 | 4.0 | 4.0 |
| 34 | 3.3 | 4.6 | 3.6 | 4.0 |
| 35 | 3.0 | 5.0 | 4.0 | 3.5 |
| 36 | 3.5 | 5.0 | 5.0 | 5.0 |
| 37 | 3.5 | 5.0 | 5.0 | 5.0 |
| 38 | 5.0 | 5.0 | 5.0 | 5.0 |
| 39 | 4.0 | 5.0 | 5.0 | 4.0 |
| 40 | 3.0 | 2.0 | 5.0 | 4.5 |
| 41 | 3.0 | 5.0 | 5.0 | 5.0 |
| 42 | 3.0 | 5.0 | 4.0 | 4.0 |
| 43 | 5.0 | 5.0 | 5.0 | 5.0 |
| 45 | 4.0 | 5.0 | 2.0 | 4.0 |
| 46 | 4.0 | 4.0 | 4.0 | 4.0 |
| 47 | 4.0 | 5.0 | 4.0 | 4.0 |
| 48 | 3.0 | 5.0 | 3.0 | 4.0 |
| 50 | 3.0 | 4.0 | 4.0 | 4.0 |
| 51 | 4.5 | 4.5 | 4.5 | 4.0 |
| 53 | 4.0 | 4.5 | 5.0 | 5.0 |
| 54 | 5.0 | 5.0 | 5.0 | 5.0 |

# Table 8: Mentor perceptions of student preparation, work ethic, and quality of deliverables in terms of their expectations for undergraduate researchers (n=48 students, rated by 63 mentors).

Ratings on a 5-point Likert scale from 1=*Well below average* to 5=*Well above average*. For students who were mentored by more than one person, an average of their mentors’ ratings was used.

|  | **Preparation** | **Work ethic** | **Quality of presentation** | **Quality of poster** |
| --- | --- | --- | --- | --- |
| Well below average  (Rating = 1.0-1.4) | 2% | 6% | 4% | 2% |
| Slightly below average  (Rating = 1.5-2.4) | 8% | 8% | 8% | 4% |
| Average  (Rating = 2.5-3.4) | 35% | 13% | 15% | 17% |
| Slightly above average  (Rating = 3.5-4.4) | 31% | 8% | 40% | 40% |
| Well above average  (Rating = 4.5-5.0) | 23% | 65% | 33% | 37% |

Note: Data are rounded to the nearest whole number.

# Table 9: Mentor rating of student progress as scientists-in-training (n=20 students rated by 29 mentors*).

Responses on a 4-point Likert scale from 1=*Very little* to 4=*Significant*. For students who were mentored by more than one person, an average of the mentors’ ratings is shown.

| **Student ID** | **Mentor(s) rating** |
| --- | --- |
| 32 | 3.0 |
| 33 | 4.0 |
| 34 | 3.0 |
| 35 | 4.0 |
| 36 | 4.0 |
| 37 | 3.5 |
| 38 | 4.0 |
| 39 | 4.0 |
| 40 | 4.0 |
| 41 | 4.0 |
| 42 | 4.0 |
| 43 | 4.0 |
| 45 | 4.0 |
| 46 | 3.0 |
| 47 | 4.0 |
| 48 | 4.0 |
| 50 | 3.0 |
| 51 | 4.0 |
| 53 | 4.0 |
| 54 | 4.0 |

*Data collected from 2018 and 2019 cohorts only
