# Supplementary material for: Early access to science research opportunities: Growth within a geoscience summer research program for community college students
Source: PLoS One. 2023 Dec 21;18(12):e0293674. doi: 10.1371/journal.pone.0293674 (PMC10734936; doi:10.1371/journal.pone.0293674)
Supplement: S3 File — (DOCX) [file pone.0293674.s003.docx]

Supporting Information (S3) for

Early access to science research opportunities: Growth within a geoscience summer research program for community college students

Christine Okochi^1 *^, Anne U. Gold^1^, Alicia Christensen^1^, Rebecca L. Batchelor^1^

^1^ Cooperative Institute for Research in Environmental Science, University of Colorado Boulder, Boulder, Colorado, United States of America

*Corresponding author

[christine.okochi@colorado.edu](mailto:christine.okochi@colorado.edu)

## **Table: Cohen’s kappa for inter-rater reliability.**

| **Codes for student data** | **Cohen's kappa** | **p-value** |
| --- | --- | --- |
| Confirmed or inspired new interest in graduate school. | 0.739 | <.001 |
| Introduced to others in the scientific community. | 0.835 | <.001 |
| Confirmed or inspired new interest in research. | 0.798 | <.001 |
| More aware of options and resources. | 0.505 | <.001 |
| Prepared for graduate school. | 0.734 | <.001 |
| Clarified field of study wanted to pursue. | 0.571 | <.001 |
| Increased confidence in abilities. | 0.626 | <.001 |
| Introduced me to a new field of study I want to pursue. | 0.731 | <.001 |
| Confirmed interest in field of study. | 0.731 | <.001 |
| Still undecided about academic/career plans. | 0.658 | <.001 |
| Prepared me for a job. | 0.658 | <.001 |
| Prepared for 4-year college. | 1 | <.001 |
| Enhanced resume. | n/a | n/a |
| Prepared for advanced coursework. | n/a | n/a |
| **Codes for mentor data** |  |  |
| All 5 codes | 0.919 | <.001 |
